# Near-source passive sampling for monitoring viral outbreaks within a university residential setting

**DOI:** 10.1017/S0950268824000190

**Published:** 2024-02-08

**Authors:** Kata Farkas, Jessica L. Kevill, Latifah Adwan, Alvaro Garcia-Delgado, Rande Dzay, Jasmine M. S. Grimsley, Kathryn Lambert-Slosarska, Matthew J. Wade, Rachel C. Williams, Javier Martin, Mark Drakesmith, Jiao Song, Victoria McClure, Davey L. Jones

**Affiliations:** 1 School of Environmental and Natural Sciences, Bangor University, Bangor, UK; 2 Data Analytics & Surveillance Group, UK Health Security Agency, London, UK; 3 The London Data Company, London, UK; 4 School of Engineering, Newcastle University, Newcastle-upon-Tyne, UK; 5 Division of Vaccines, Medicines and Healthcare Products Regulatory Agency, Hertfordshire, UK; 6 Communicable Disease Surveillance Centre, Public Health Wales, Cardiff, UK; 7 Food Futures Institute, Murdoch University, Murdoch, WA, Australia

**Keywords:** enteric viruses, public health, passive sampler, respiratory viruses, university campus

## Abstract

Wastewater-based epidemiology (WBE) has proven to be a powerful tool for the population-level monitoring of pathogens, particularly severe acute respiratory syndrome coronavirus 2 (SARS-CoV-2). For assessment, several wastewater sampling regimes and methods of viral concentration have been investigated, mainly targeting SARS-CoV-2. However, the use of passive samplers in near-source environments for a range of viruses in wastewater is still under-investigated. To address this, near-source passive samples were taken at four locations targeting student hall of residence. These were chosen as an exemplar due to their high population density and perceived risk of disease transmission. Viruses investigated were SARS-CoV-2 and its variants of concern (VOCs), influenza viruses, and enteroviruses. Sampling was conducted either in the morning, where passive samplers were in place overnight (17 h) and during the day, with exposure of 7 h. We demonstrated the usefulness of near-source passive sampling for the detection of VOCs using quantitative polymerase chain reaction (qPCR) and next-generation sequencing (NGS). Furthermore, several outbreaks of influenza A and sporadic outbreaks of enteroviruses (some associated with enterovirus D68 and coxsackieviruses) were identified among the resident student population, providing evidence of the usefulness of near-source, in-sewer sampling for monitoring the health of high population density communities.

## Key findings

- Wastewater surveillance is feasible for small, high-density communities
- Passive sampling is a low-cost, simple approach for building-level wastewater monitoring
- Enteric and respiratory pathogens can be monitored in sewers quantitatively via quantitative polymerase chain reaction (qPCR)
- Viral variants/strains can also be identified in sewers via sequencing

## Introduction

The causative agent of the COVID-19 pandemic, severe acute respiratory syndrome coronavirus 2 (SARS-CoV-2), is now the most closely monitored virus worldwide [1]. Surveillance in most countries has combined the use of conventional clinical sampling alongside the monitoring of viral levels in municipal wastewater [2, 3]. Wastewater-based epidemiology (WBE) is a powerful and relatively cost-effective public health tool, providing complementary data to traditional clinical surveillance metrics [4]. Due to the success of WBE in providing public health insights for SARS-CoV-2, further applications to monitor additional human pathogens to support a wide range of public health uses are being explored (e.g. influenza viruses, antimicrobial-resistant bacteria) [5, 6]. Efforts have also focused on viruses transmitted via the faecal–oral route, such as norovirus, enteroviruses including poliovirus, and adenoviruses [7]. Often sewage monitoring has focused on effective ways to eliminate viruses from wastewater to protect the wider environment via treatment technologies [8]; however, there is an increasing emphasis on the utility of WBE for public health surveillance. In recent years, this pathogen-specific, targeted approach has been complemented by untargeted monitoring of viruses in wastewater and the wider environment (metaviromics) to evaluate the diversity of human and zoonotic viruses, which may include new and emerging strains [9].

The importance of WBE for unbiased, population-level viral monitoring is now well-established, and a wide range of approaches have been rapidly developed to facilitate the collection of representative samples, both temporally and spatially. These have largely focused on the collection of samples from centralized wastewater treatment plants (WWTPs) and from sub-catchments within the sewer network [10–12]. However, there has also been strong interest in monitoring SARS-CoV-2 near the source of shedding, where there is perceived to be a high risk of disease transmission (e.g. university accommodation and healthcare facilities) [13, 14].

To date, most of the research focusing on SARS-CoV-2 in wastewater has employed single samples taken daily, either via grab sampling or using autosamplers to collect composite samples throughout the day [15]. However, for near-source sampling, both grab and composite liquid samples can be difficult to acquire due to sampling challenges that limit the ability to use autosamplers or take grab samples, such as low and intermittent wastewater flow rates upstream in the sewer network and poor accessibility to sewer pipes at these locations. Furthermore, grab samples may be unreliable due to the higher temporal variation in bathroom usage habits that prevents representative samples from being obtained. Autosamplers provide a time- or flow-integrated sampling approach but can be costly and difficult to deploy rapidly, especially for near-source sampling where sewage flow rates are low and the risk of blockages is greater. The lack of a local power supply and the risk of equipment theft are also a concern with autosamplers. Other methods for sampling wastewater near source are typically needed.

Passive sampling provides an alternative solution in scenarios where the collection of grab or composite samples is not appropriate, or when there are fiscal constraints. Passive sampling stems from previous studies, which deployed cotton swabs into the wastewater stream to capture viruses as they passed through the sewer [16]. Most passive samplers are made from materials that have electrostatically charged surfaces to which viral particles become attracted and are retained [17]. They can also physically trap particulate matter to which the viruses may be bound [18]. Furthermore, in terms of viral recovery, passive samplers have been shown to outperform grab samples, due to the latter being highly sensitive to temporal changes in viral load. The use of passive sampling for near-source monitoring of SARS-CoV-2 has been used as evidence to avert outbreaks in student populations [19]; however, its feasibility for monitoring other viruses has not been investigated to date.

In this study, we assessed the use of cotton tampon-based passive samplers for near-source sampling of large student residential blocks and recreational areas at Bangor University, UK. We focused on SARS-CoV-2 and other viruses of public health concern, such as influenza viruses and enteroviruses, including the emerging enterovirus D68 variant. The overall aim was to assess the usefulness of this sampling approach for the monitoring of near-source environments.

## Methods

### Sample sites

**Figure 1.**
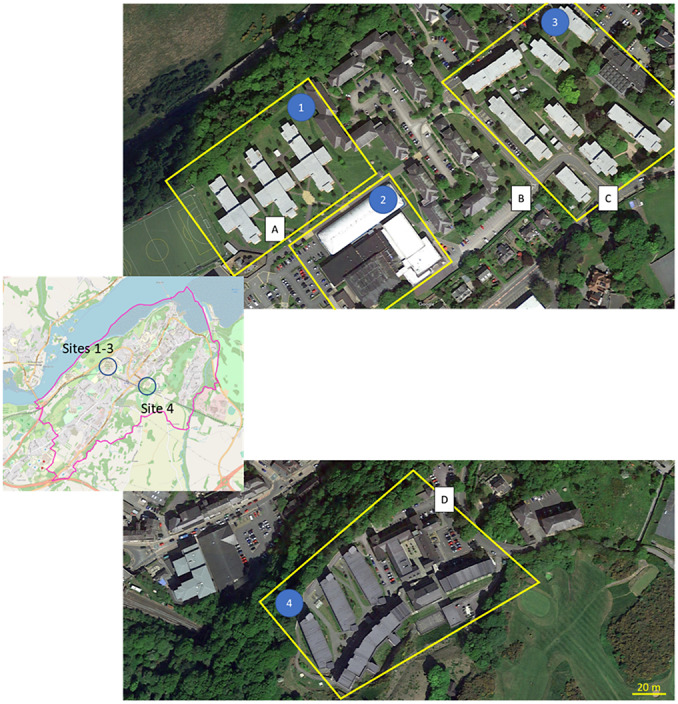
Aerial photograph showing the location of the four sampling sites. Letters indicate the approximate location of the manholes where sampling took place (corresponding to the area numbers). Ffriddoedd site – western residential blocks (Site 1), Ffriddoedd site – Brailsford sports facility (Site 2), Ffriddoedd site – eastern residential blocks (Site 3), and St Mary’s residential blocks (Site 4). Bangor map: © OpenStreetMap.

A viral monitoring trial was undertaken at three sites on the main Bangor University campus located in Bangor, Gwynedd, UK (Figure 1). Sampling was undertaken in two independent student residential areas, namely the Ffriddoedd Site (1800 residents; 53°13′31.71‘’N, 4°8′24.11‘’W, Sites 1 and 3) and the St Mary’s Site (600 residents; 53°13′20.34‘’N, 4°7′45.80‘’W, Site 4), and at one communal indoor sports facility (Canolfan Brailsford Sports Centre; 53°13′28.91‘’N, 4°8′29.75‘’W, Site 2). The sports facility was included to gather data on asymptomatic cases, assuming that people feeling unwell would not visit the centre. Near-source sample points were selected based on the following criteria: (i) to be able to capture the main sewer flow from residential blocks and the university sports facility; (ii) the maintenance holes for sewer access were located on Bangor University property, thus providing unlimited access; and (iii) the network was exclusive to the targeted buildings (i.e. no other housing fed into the sewer network).

### Passive sampler type and deployment

All the monitoring was undertaken with Tampax Compak Pearl Super cotton-based tampons (Procter & Gamble Inc., USA). The size, availability, and reproducibility of the passive samplers were a factor in their choice. The tampons were loaded in a perforated plastic holding device that continuously exposed the tampon to flowing wastewater (‘torpedo device’) [16, 20]. The device holding the tampon was then suspended in the middle of the sewer on a string, which was then tethered to the manhole to prevent the loss of the passive sampler.

### Sample collection

Sampling was conducted between 25 October 2021 and 26 January 2022 with a four-week break between 15 December and 9 January when the students were absent from campus. Each Monday between 08.00 h and 09.00 h, passive samplers were deployed in the sewer at the selected sites (a to d, Figure 1) and then collected between 14.00 h and 15.30 h on the same day (PM samples; ca. 7-h deployment). At this time, another passive sampler would be deployed and collected the following morning (AM samples; ca. 17-h deployment). This was repeated each day from Monday to Thursday. On Fridays, no passive sampler would be deployed in the afternoon. No sampling was conducted over the weekend. Once samples were retrieved from the sewer, they were placed into a sealed polyethylene bag and stored in a cool box with ice packs until their arrival to the laboratory (within 2 km), where they were transferred to 4 °C storage. Overall, 296 samples were collected during the study.

### Sample processing

#### Viral recovery

To recover viruses from the passive samplers, 20 ml of sterile phosphate-buffered saline (PBS) was added to the sample bag to saturate the passive sampler, which was massaged lightly by hand for 2 min. The passive sampler was squeezed to release all liquid, the corner of the bag was cut, and the liquid was poured into a 50-ml sterile centrifuge tube. Samples were then centrifuged at 10000x*g* for 10 min, to precipitate larger particulates. The supernatant was transferred to a sterile 50-ml centrifuge tube, and the pellet was discarded. At this stage, an aliquot of the double-stranded (ds) ribonucleic acid (RNA) *Pseudomonas* phage Phi6 (10^6^–10^7^ genome copies (gc)/sample) was added as a process control to the sample. Each sample was mixed with polyethylene glycol 8000 (PEG8000) and NaCI to reach the final concentration of 10% and 2%, respectively. The tubes were inverted several times to mix and then stored at 4 °C for 16 h. Subsequently, the samples were precipitated via centrifugation at 10000x*g* at 4 °C for 30 min. The supernatant was discarded, and the pellet was resuspended in 800 μl of NucliSens® lysis buffer (bioMérieux SA, France).

#### Total viral nucleic acid extraction

The viral RNA and deoxyribonucleic acid (DNA) were then extracted using the NucliSens extraction reagents (BioMérieux, France) on the KingFisher 96 Flex Automated Purification System (Thermo Scientific, USA) using our previously published protocol [21].

#### Viral quantification

Quantitative polymerase chain reaction (qPCR) was applied for the detection and quantification of faecal indicator virus crAssphage as described previously [21]. We used duplex reverse transcription qPCR (RT-qPCR) assays to detect and quantify the SARS-CoV-2 N1 gene together with Phi6, SARS-CoV-2 mutations pertaining to variants of concern (VOCs Beta–Gamma and Delta–Kappa), influenza A and B viruses, *Enterovirus* spp., and enterovirus D68, as detailed in Supplementary Tables S1 and S2, using existing protocols [22, 23]. All reactions were run on a QuantStudio Flex 6 (Applied Biosystems Inc., Waltham, USA), at a reaction volume of 20 μl.

For the SARS-CoV-2 Omicron variant, a novel assay was developed with primers and probes targeting the S371L, S371F, S373P, and S375F mutations of the S gene of the BA.1 and BA.2 lineage genomes. For sensitivity assessment, the limit of detection (LOD) and limit of quantification (LOQ) were determined as described previously [24]. For specificity, the assay was tested on a dilution series (1–10^5^ copies/μl) of genomic RNA of the SARS-CoV-2 Wuhan strain and the Beta, Gamma, Delta, and Kappa variants.

In all (RT)-qPCR assays, samples were run in duplicate, against a single-stranded (ss)RNA (SARS-CoV-2 N1, Phi6, influenza A and B), synthetic genome (SARS-CoV-2 VOCs, enterovirus, and D68; Twist Bioscience Ltd., USA), or plasmid DNA (crAssphage, norovirus GII; [24, 25]) standard curve dilution series of the target sequence in the range of 1–10^5^ copies/μl. PCR non-template controls (molecular-grade water) were used to determine the absence of contamination during the PCR set-up. For RNA targets (Supplementary Table S2) and crAssphage, the TaqMan Fast Virus 1-Step Master Mix (Applied Biosystems Inc., USA) and the QuantiNova Probe PCR Kit (Qiagen, Germany) were used, respectively, as detailed in Supplementary Table S2.

All collected samples were tested for crAssphage, Phi6, SARS-CoV-2 (N1), the Delta and Gamma variants, influenza A/B viruses, enteroviruses, and enterovirus D68. The samples collected in October–December 2021 were tested for the SARS-CoV-2 Beta and Kappa variants. The samples collected in December 2021 and January 2022 were tested for norovirus GII, and the samples collected in January were subject to SARS-CoV-2 Omicron variant RT-qPCR.

### SARS-CoV-2 next-generation sequencing (NGS)

To test whether the viral RNA obtained from passive samples can be used to detect and sequence variants of interest, 48 samples collected between 22 November 2021 and 22 January 2022 underwent whole-genome sequencing. RNA extracts were cleaned following an optimized protocol for sequencing RNA from wastewater [26]. Briefly, RNA was purified by using a magnetic bead clean-up of 1.8X Mag-Bind Total NGS beads (Omega Bio-Tek, USA). The circular DNA (cDNA) was synthesized with the LunaScript RT SuperMix Kit (New England Biolabs, UK) before sequencing libraries were prepared using the EasySeq RC`PCR SARS-CoV-2 whole-genome sequencing kits (NimaGen, The Netherlands). The final library was spiked with PhiX (an adapter-ligated library supplied by Illumina and used as a control in Illumina sequencing runs) and ran on a NextSeq 1000 system using a P1 kit (2x150 bp) following concentration loading guidelines provided by Illumina.

### Enterovirus sequencing

Amplicon sequencing using the MinION platform (Oxford Nanopore Technologies, UK) was used for enterovirus sequencing, as previously described by the Polio Sequencing Consortium [27]. In brief, the samples positive for *Enterovirus* spp. or enterovirus D68 using RT-qPCR were subject to RT-PCR using the SuperScript™ III One-Step RT-PCR System (Thermo Fisher, USA) with the panEV primers targeting a ~ 3.9 kb region of the enterovirus genome [28]. Next, nested PCR was performed on the RT-PCR-positive samples using three reactions containing a combination of the Sabin1-VP1 forward and reverse primers (Reaction A) [27], the Y7/Q8 primers (Reaction B) [29], and the Sabin1-VP1F/Q8 primers (Reaction C), all targeting the VP1 gene. Of the 53 samples, seven were positive with nested PCR. The PCR products were purified using the AMPure XP beads (Beckman Coulter Life Sciences, USA) followed by library preparation with the V14 SQK-NBD114 Ligation Sequencing Kit (Oxford Nanopore Technologies, UK) following the manufacturer’s instructions. Sequencing was performed on the MinION Mk1C sequencer using R10 flow cells (Oxford Nanopore Technologies, UK).

### Data analysis

The qPCR quality control (QC) was performed using the QuantStudio Real-Time PCR Software v1.7 (Applied Biosystems, Inc., USA). The results were expressed as gc/μl RNA extract and were transformed into gc/sampler. Samples with at least one replicate with a Ct value <40 and a concentration of 0.25 gc/μl were considered positive. No normalization was performed due to the lack of water flow data. When Phi6 process control virus recovery was lower than 0.1%, the 2x diluted samples were quantified again. CrAssphage and SARS-CoV-2 gene copy concentrations were compared using Spearman’s correlation, Wilcoxon’s rank-sum exact, and the Kruskal–Wallis tests using Statistical Package for the Social Sciences (SPSS) V.27 (IBM, SPSS Inc., USA).

Variant sequencing data were processed using the single-nucleotide variant (SNV) frequency estimation and depth-weighted demixing tool, Freyja v1.2.1 [30], to estimate the relative abundance of SARS-CoV-2 lineages from the sequencing of mixed-lineage virus samples in wastewater. QC was carried out by the Nextflow implementation of the Artic pipeline (https://github.com/connor-lab/ncov2019-artic-nf). Samples passed QC when >50% of the reference sequence bases were identified in >10 reads; if these criteria were not met, then the samples failed QC [31].

For enterovirus sequencing, the Fastq files were mapped against a custom database of polio and non-polio enteroviruses from the National Center for Biotechnology Information (NCBI) Nucleotide (https://www.ncbi.nlm.nih.gov/nuccore/) and the National Institute of Allergy and Infectious Diseases (NIAID) Virus Pathogen Database and Analysis Resource using Geneious Prime v2023.0.4 (Biomatters, New Zealand). Mapping results in read number < 30 were excluded from the analysis.

### COVID-19 case numbers

During the study period, students living at university accommodation were urged to get tested for SARS-CoV-2 when they expressed any typical symptoms of COVID-19 (e.g. coughing, headache, loss of smell, nausea). Only seven students living at Site 3 were reported as being COVID-19-positive (Table 1). No positive cases were reported from residential Sites 1 and 4. Data were not available for testing of sports facility users and staff at Site 2.

**Table 1.** Testing dates and number of students tested positive for COVID-19 during the study period at Site 3

Date of test	Form of test	Number of students testing positive
14 November 2021	Lateral flow	1
30 December 2021	PCR	1
3 January 2022	Lateral flow	1
14 January 2022	Lateral flow/PCR	2
20 January 2022	Lateral flow	1

## Results

Overall, 274 samples were spiked with Phi6 phage and 87% of the spiked samples were positive for Phi6 resulting in 0.002%–45% recoveries (mean of 1%). The dilution of the sample had no considerable effect on recovery, suggesting that the low recovery was not a result of RT-qPCR inhibition but due to incomplete recovery during sample processing.

All target viruses, except influenza B virus, were detected in student accommodation wastewater using passive samplers via qPCR-based detection and quantification (Table 2). Most samples (99% of the total) also tested positive for the faecal indicator virus crAssphage, suggesting that the samplers successfully collected faecal matter. We found no difference in crAssphage detection rates and concentrations between AM and PM samples and among sites. A weak negative correlation was observed between crAssphage and SARS-CoV-2 (Spearman’s rho = −0.139, *p* = 0.017), and a strong positive correlation between crAssphage and influenza A virus titres was found (Spearman’s rho = 0.523, *p* < 0.001; Supplementary Table S3).

**Table 2.** Detection rates (*n*) of target virus RNA segments in wastewater collected from student accommodation and associated sports facility, using passive samplers recovered from the sewer either in the morning (AM) or in the afternoon (PM) over 38 independent sampling days

			SARS-CoV-2 variants of concern								
Site	CrAssphage	SARS-CoV-2	Beta	Delta	Gamma	Kappa	Omicron	EV	EV-D68	NoV GII	FluA
Site 1	100% (74)	49% (74)	0% (54)	15% (74)	4% (68)	0% (59)	67% (15)	4% (73)	10% (73)	0% (19)	31% (74)
Site 1 AM	100% (31)	45% (31)	0% (26)	13% (31)	3% (29)	0% (26)	60% (5)	7% (30)	3% (30)	0% (7)	32% (31)
Site 1 PM	100% (43)	51% (43)	0% (33)	16% (43)	5% (39)	0% (33)	70% (10)	2% (43)	14% (43)	0% (12)	30% (43)
Site 2	97% (74)	65% (74)	2% (59)	42% (74)	9% (68)	0% (59)	53% (15)	24% (74)	16% (74)	0% (19)	28% (74)
Site 2 AM	97% (31)	71% (31)	0% (26)	58% (31)	10% (29)	0% (26)	40% (5)	26% (31)	16% (31)	0% (7)	29% (31)
Site 2 PM	98% (43)	60% (43)	3% (33)	30% (43)	8% (39)	0% (33)	60% (10)	23% (43)	16% (43)	0% (12)	28% (43)
Site 3	100% (73)	61% (73)	3% (59)	30% (73)	4% (67)	0% (59)	36% (14)	1% (73)	8% (73)	0% (18)	34% (73)
Site 3 AM	100% (30)	67% (30)	0% (25)	40% (30)	0% (28)	0% (25)	40% (5)	0% (30)	7% (30)	0% (7)	43% (30)
Site 3 PM	98% (43)	58% (43)	6% (33)	24% (42)	8% (38)	0% (33)	33% (9)	2% (42)	10% (42)	0% (11)	29% (42)
Site 4	95% (73)	68% (73)	7% (59)	38% (73)	13% (67)	3% (59)	57% (14)	12% (73)	8% (73)	50% (18)	26% (73)
Site 4 AM	90% (30)	60% (30)	8% (26)	43% (30)	18% (28)	4% (26)	73% (4)	17% (30)	7% (30)	50% (6)	33% (30)
Site 4 PM	98% (43)	74% (43)	6% (33)	35% (43)	10% (39)	3% (33)	50% (10)	9% (43)	9% (43)	50% (12)	21% (43)

FluA, influenza A; EV, enterovirus; EV-D68, enterovirus D68; NoV GII, norovirus GII.

### SARS-CoV-2 detection

**Figure 2.**
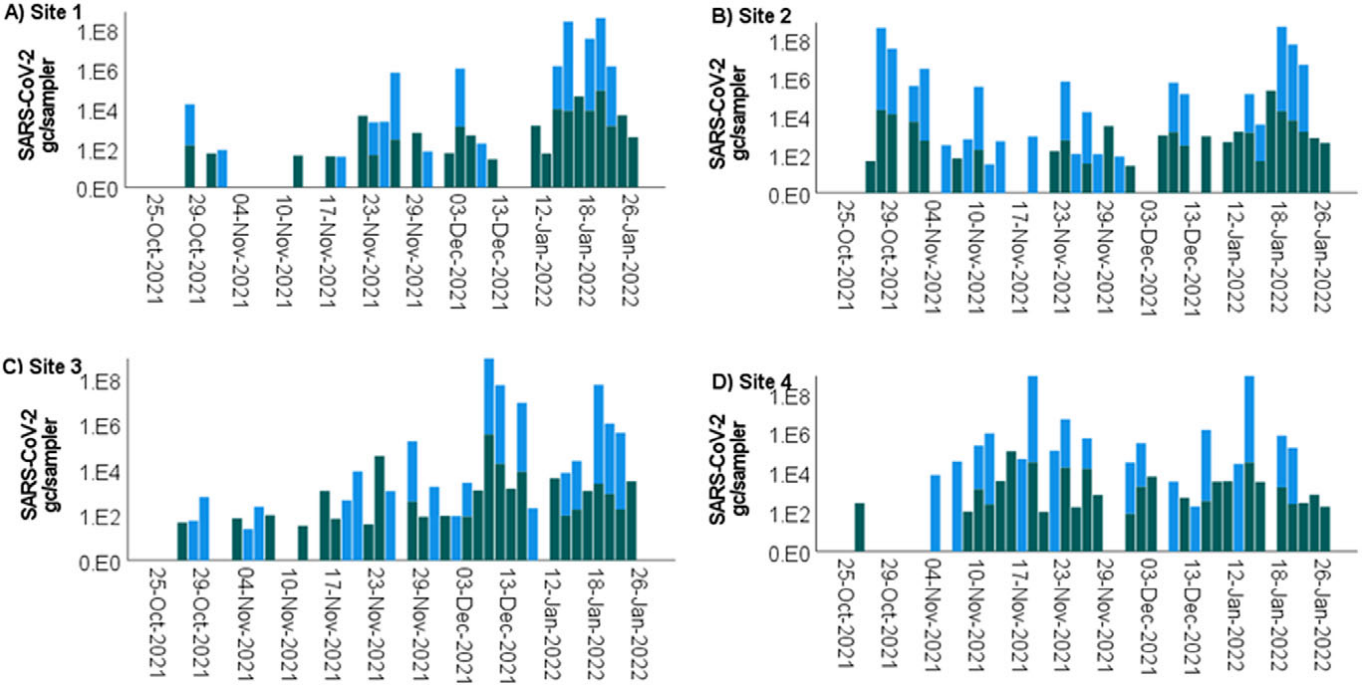
SARS-CoV-2 N1 concentration (genome copies (gc)/sampler) in passive samplers deployed in the sewer network in a university residential setting at (a) Site 1: Ffriddoedd West residential block, (b) Site 2: sports facility – Brailsford, (c) Site 3: Ffriddoedd East residential block, and (d) Site 4: St Mary’s residential block. The passive samplers were collected in the AM (blue) and in the PM (green). The absence of bars indicates that the sample was negative for SARS-CoV-2. A four-week break in sampling was implemented between 15 December and 9 January when the students were absent from campus.

SARS-CoV-2 was detected using the passive samplers at all four campus sampling locations representing 45% to 74% of samples (Table 2). At Sites 1 and 4, viral detection rates were higher during the PM samples, and at Sites 2 and 3, the virus was more abundant during the AM sampling (Table 2). In addition, the SARS-CoV-2 viral loads detected in the AM and PM passive samplers showed variation (Figure 2); however, no statistically significant differences were identified (*p* > 0.05). Significant differences in SARS-CoV-2 concentrations were observed among the four sites (Kruskal–Wallis test, *p* = 0.018). The highest concentrations over the study period were observed at Site 4 (1.2 x 10^4^ gc/sampler), followed by Site 3 (1.1 x 10^4^ gc/sampler) and Site 2 (7.67 x 10^3^ gc/sampler). The lowest SARS-CoV-2 concentrations were detected at Site 1 (5.8 x 10^3^ gc/sampler).

Due to the low number of clinically confirmed COVID-19 cases recorded during the study period (Table 1), no correlation analysis between case numbers and wastewater SARS-CoV-2 data was possible. However, the SARS-CoV-2 virus concentrations in wastewater showed a good visual correlation in weekly COVID-19 cases monitored among inpatients in the local hospital (Supplementary Figure S1). The case number is a sensible proxy for the level of local community infection for the study period due to the absence of community testing.

### Detection of SARS-CoV-2 variant-specific mutations

The samples were also screened for SARS-CoV-2 spike protein mutations indicative of VOCs (Supplementary Table S1). The mutation 156-157DEL associated with the Delta VOC was frequently detected at all sampling sites (Table 2) with a concentration range of 3 to 4.11x10^4^ gc/sampler (Supplementary Figure S2). The mutation K417T specific to Gamma VOC was also detected at all sites; however, the detection rates (7%) were lower than those observed for the Delta-specific mutation and had a wider range of concentrations (6.07x10^3^ to 1.24x10^5^ gc/sampler). The mutation K417N associated with the Beta VOC was sporadically detected at Sites 2–4 (5.30x10^2^ to 1.35x10^4^ gc/sampler), whereas the Kappa VOC mutation E154K was only detected at Site 4 (62–1.79x10^3^ gc/sampler). Due to their low abundance, RT-qPCR assays targeting the mutations associated with the Beta and Kappa variants were terminated in December 2021.

Due to the rapid emergence of the Omicron variant at the end of 2021, the samples collected in January 2022 were tested using a novel RT-qPCR assay targeting the S371L, S371F, S373P, and S375F mutations (Supplementary Table S1). The assay was highly sensitive with LOD of 2.11 gc/μl RNA extract and with LOQ of 5.23 gc/μl RNA extract with no cross-reactivity with the Wuhan SARS-CoV-2 strain or with Beta, Gamma, Kappa, or Delta variants. The Omicron variant was highly abundant at all sites with concentrations between 43 and 4.32x10^4^ gc/sampler (Supplementary Figure S2).

**Figure 3.**
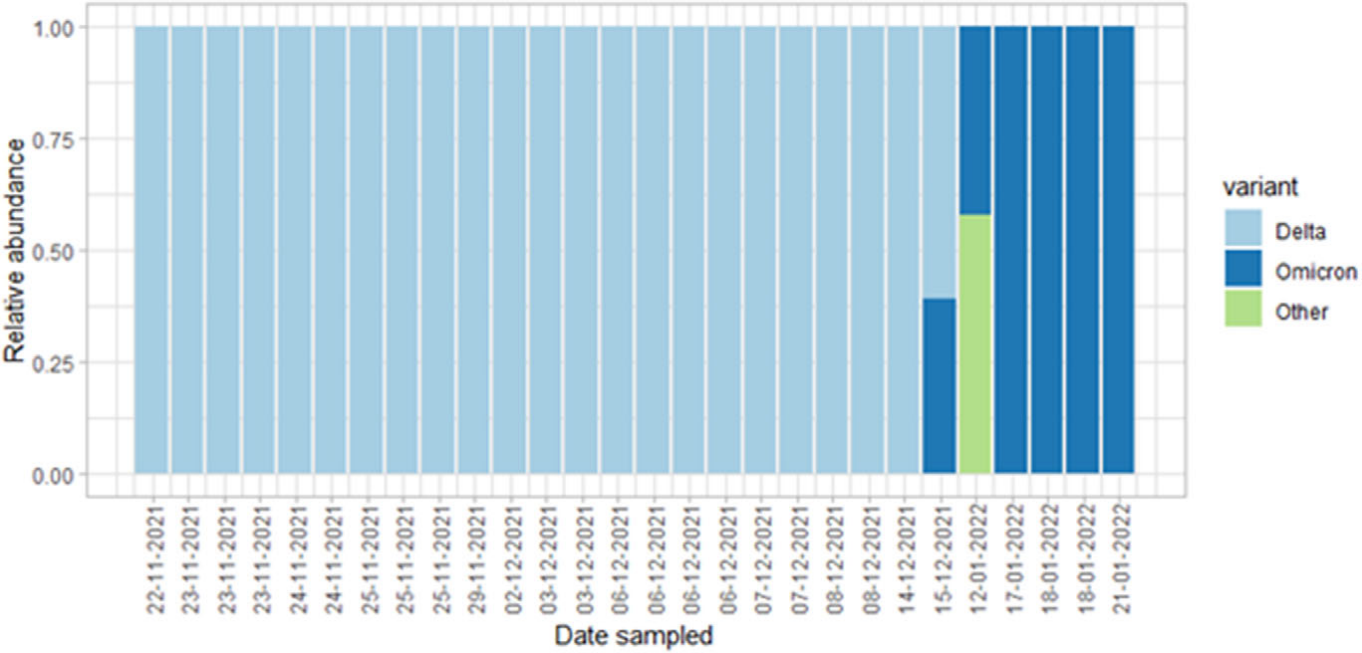
SARS-CoV-2 variants of concern identified in student accommodation wastewater samples between November 2021 and January 2022 using sequencing data. Proportion of sequence reads assigned to each variant using Freyja program; variant ‘other’ represents lineage abundances that do not fall into the World Health Organization grouping.

The sequencing results show that 28 out of 48 samples passed the QC, and 20 failed. In mid-November, Delta was the only variant detected in the samples. By mid-January, Omicron was detected at 99% abundance and was present in each subsequent sample collected until the end of January (Figure 3), in agreement with the results of the RT-qPCR assays on variant-specific mutation (Supplementary Figure S2). This provides evidence that sequence data from near-source passive samplers can be used for the detection of novel and emerging VOCs.

### Influenza A virus detection

**Figure 4.**
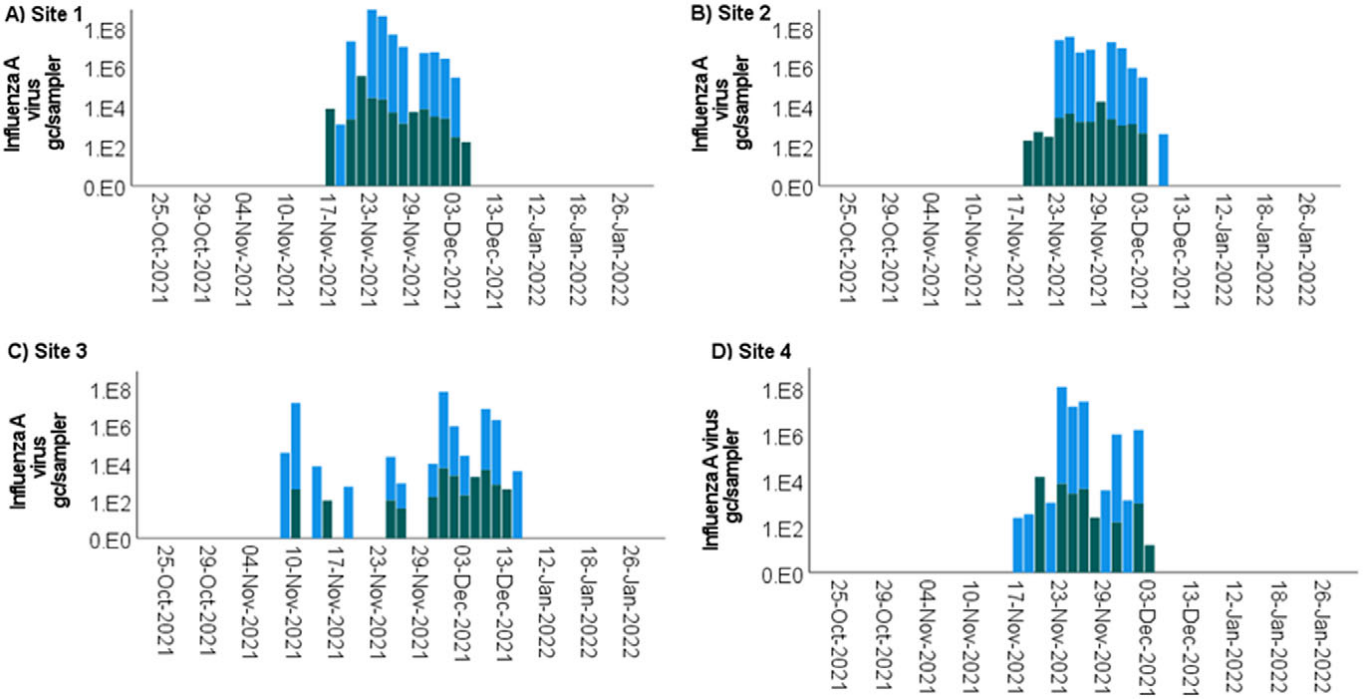
Influenza A virus concentration (gc/sampler) in passive samplers deployed in the sewer network in a university residential setting at (a) Site 1: Ffriddoedd West residential block, (b) Site 2: sports facility – Brailsford, (c) Site 3: Ffriddoedd East residential block, and (d) Site 4: St Mary’s residential block. The passive samplers were collected in the AM (blue) and in the PM (green). The absence of bars indicates that the sample was negative for SARS-CoV-2. A four-week break in sampling was implemented between 15 December and 9 January when the students were absent from campus.

Influenza A virus was detected at all four sites between 17 November and 13 December 2021 (Figure 4). Overall, viral loads were detected with concentrations up to 3.51x10^5^ gc/sampler for all samples. Higher viral concentrations were observed in the AM samples compared with the PM samples; however, the detection rates were similar.

### Enterovirus detection

**Figure 5.**
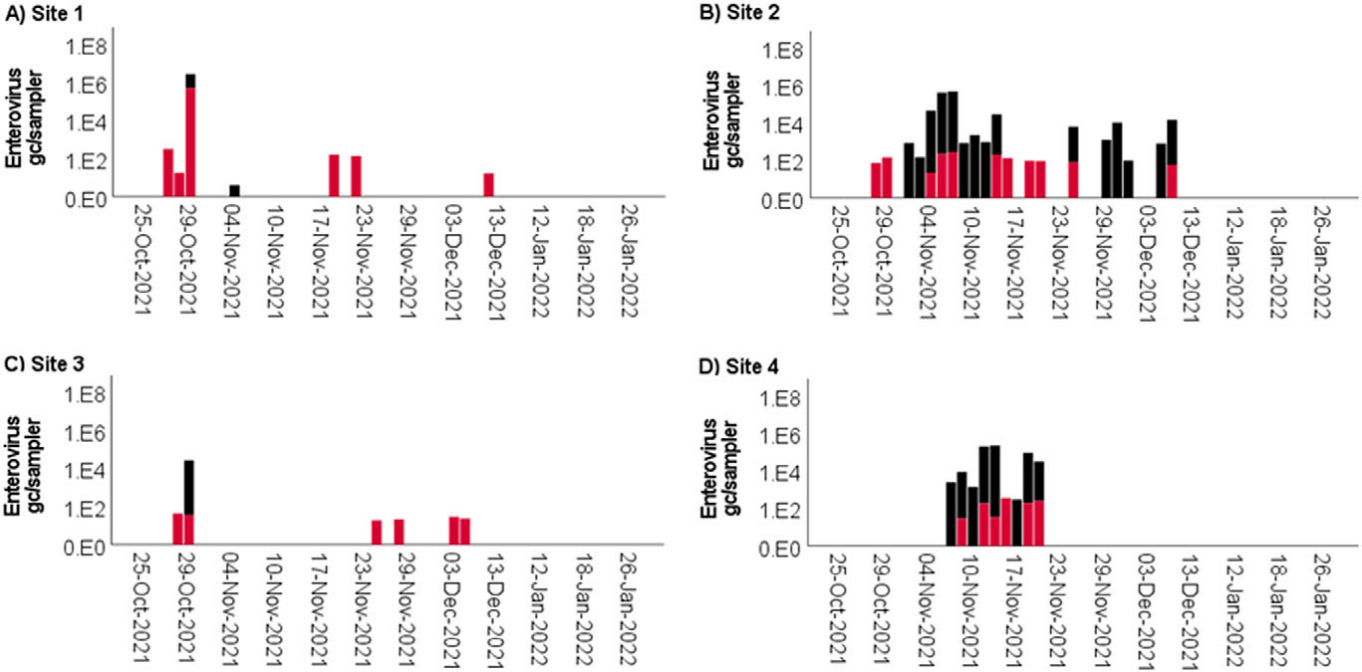
Concentration (gc/sampler) of *Enterovirus* spp. (black) and enterovirus D68 (red) in wastewater at (a) Site 1, (b) Site 2, (c) Site 3, and (d) Site 4 using passive sampler devices. A four-week break in sampling was implemented between 15 December and 9 January when the students were absent from campus.

*Enterovirus* spp. was initially detected using RT-qPCR in samples from Sites 1 and 3 at the end of October; however, these proved to be isolated incidences. Enterovirus was frequently detected at Sites 2 and 4 in November and at Site 2 in early December 2021 (Figure 5). In this data set, enterovirus loads did not exceed 10^4^ gc/sampler except for one sample from Site 1, which had >10^6^ gc/sampler virus concentration in one sample (Figure 5). No significant difference in viral detection rates and concentrations was observed during the different sampling times (*p* > 0.05).

Enterovirus D68 was also detected using RT-qPCR in the passive samplers, often at a viral load of <10^3^ gc/sampler, which may indicate few cases were present (Figure 5). Enterovirus D68 was detected at Sites 1–3 in late October and at all sites in November and early December 2021. More samples collected in the afternoon were positive for enterovirus D68 than those collected in the morning (11.6% vs. 14.6%), but no differences in concentrations were observed (*p* > 0.05). The *Enterovirus* spp. and enterovirus D68 concentrations showed a positive correlation (rho = 0.351, *p* < 0.001); however, in some cases, enterovirus D68 was detected in samples that tested negative for *Enterovirus* spp. (Figure 5), suggesting the latter assay is less sensitive.

The samples positive for *Enterovirus* spp. and/or enterovirus D68 were also subject to MinION amplicon sequencing. Of the seven PCR-positive samples, coxsackieviruses were detected in six samples. Strain A20 was detected in five samples, A19 in three samples, and A15 in one sample. Read numbers were < 90 in all but one case (Table 3). Enterovirus D68 was not detected using sequencing in any of the samples as the PCRs used in this study do not amplify the D68 strain.

**Table 3.** Taxonomic classification of MinION read in the seven samples subject to enterovirus amplicon sequencing

Sample	Coxsackievirus		
	A15	A19	A20
Site1_29/10/2021_AM	–	39	38
Site2_12/11/2021_AM	–	–	–
Site4_19/11/2021_PM	–	–	37
Site2_30/11/2021_AM	–	–	39
Site2_01/12/2021_PM	–	83	35
Site3_06/12/2021_AM	47	49	–
Site2_06/12/2021_AM	–	–	7,055

## Discussion

Low-cost passive samplers have demonstrable value when used to monitor SARS-CoV-2 in a range of environments, including near-source wastewater testing [16, 19, 32–34]. In this study, we explored the usefulness of passive samplers in a near-source setting for the simultaneous detection of different viral pathogens, including SARS-CoV-2 and its variants, influenza virus, and enteroviruses. For viral recovery from passive samplers, we first eluted viruses from the samplers using PBS and then used the traditional PEG precipitation for concentrating viruses in the eluent clarified from solids. These methods have been shown to be effective, simple, and affordable for WBE applications [17, 35, 36]. Although viral recovery has not been measured in most previous studies, the limited data suggest that 0–8% of viruses may be recovered from passive samples [35, 36], which is in line with the recovery rates we noted for Phi6 in the current study.

Overall, we conclude that these easy-to-deploy near-source passive samplers can be used successfully and that the data obtained are useful for a more holistic assessment of the health status of small, high-density populations. The cost of an individual, tampon-based passive sampler and reusable holding device also makes their deployment and subsequent recovery very cost-effective (<£1/sample), relative to autosampler devices. The latter are also prone to blockages and missed samples at near-source settings due to sporadic wastewater flow, factors that were not experienced with the passive samplers deployed in this study.

The design of a sampling regime is important to consider when conducting passive sampling as fluctuations in virus concentrations were detected in the AM and PM for all viral targets (SARS-CoV-2, influenza A, and *Enterovirus* spp., including enterovirus D68). This may be due to the differences in exposure times to the passive sampler devices. The PM samplers had less time in the sewer (7 h), while the AM samplers had a longer residence time (17 h). It has previously been identified that tampon-based passive samplers may saturate over time and are ideally suited to short exposure periods [18, 33]. It is possible, therefore, that 17 h may be excessive exposure time in the sewer. Alternatively, the results could also be attributed to the bathroom usage habits of students (i.e. some students may preferentially defecate in the morning and others at night); however, behavioural studies would be needed to confirm this.

One of the major issues with passive samplers is the lack of validated normalization factors. Although crAssphage can be an excellent indicator for the presence of faecal material, not all individuals shed crAssphage and those that do may shed the virus at different rates [37]. Another indicator, pepper mild mottle virus, also has its limitations as its presence in faecal matter is dependent on diet. It is possible that other microbial indicators, such as faecal coliforms, could be used; however, the rates of viral and bacterial adherence (i.e. binding mechanism, retention capacity) on the passive sampler material are likely to be different from the target viruses, preventing their use as a normalization factor. While inorganic tracers (e.g. NH_4_^+^, PO_4_^3−^, electrical conductivity) are frequently used for WBE in large communities, their use for near-source data normalization may be problematic as these are found in both urine and faecal material, while viruses are predominantly excreted in the latter. The number of people residing in the buildings or the volume of water used may also be used as a population proxy; however, those data were not available for the current study. Together, the lack of a robust normalization indicator makes data generated from passive samplers semi-quantitative, but still highly suited to the early identification of outbreaks and their subsequent disappearance. The rate of viral shedding in faecal matter and urine at different stages of infection and across individuals is unknown for many viruses; therefore, more studies are required to investigate this. This information will also aid in the flow normalization of any viral data generated.

Despite these limitations, we clearly show in this study that different lineages of SARS-CoV-2 VOCs can be detected by either qPCR or NGS from passive sampler materials. The NGS data were able to determine variants present in a sample with confidence, therefore highlighting the potential of samples obtained by passive samplers for downstream molecular applications. We successfully obtained sequencing data for all samples collected, although QC pass rates at the data analysis stage were 58%. Failing to pass QC is likely due to poor sample quality resulting in low mapping rates, the cause of which needs further investigation. Previous studies using passive samplers for near-source wastewater monitoring also successfully sequenced the Delta AY.103 and the Omicron BA.1 and BA.2 variants with similar QC pass rates as established in the current study [38, 39]. Furthermore, metaviromics approaches have also been successfully applied to passive sampler extracts for the identification of human pathogen viruses in wastewater [40], suggesting that the sample quality is sufficient for a range of genomics applications.

The SARS-CoV-2 results of this study showed a good visual correlation with local COVID-19 case numbers among inpatients in the local hospital, suggesting that the approach used was suitable for WBE applications. Furthermore, the use of near-source passive samplers provided evidence of several outbreaks of influenza A within the student residences. Furthermore, the influenza A virus was detected over 4-day periods, suggesting that the duration of viral shedding may be limited to a few days after infection. It also indicates that the mitigation measures put in place to prevent the in-house spread of SARS-CoV-2 may have also helped curtail the spread of influenza within the student residences (e.g. social distancing, media campaigns, isolation when respiratory symptoms occurred, face coverings). This hypothesis is consistent with the unusually low number of influenza cases seen in England and Wales during the COVID-19 pandemic and within the reporting window studied here [41]. The lack of influenza B virus in our study is also in agreement with the overall low national prevalence of the reported clinical cases [41]. Previous studies at centralized WWTPs have indicated that influenza viruses can stem from avian sources [42]. Due to the very near-source nature of the sampling campaign and the separation of sewerage from other drainage systems (e.g. rainfall collection), we are confident that no bird faecal material would enter the sewers where the samples were collected.

While clear outbreaks of influenza have been shown, we also detected sporadic outbreaks of enteroviruses, further highlighting that the near-source passive sampling approach represents a powerful tool to assess the health status of a localized population beyond COVID-19. The quantitative data suggested more cases at Sites 2 and 4 than at the other two locations, which may have been due to different student numbers at the sites. Only seven samples were positive using sequencing, which may be attributed to RNA decay, due to the sequencing being performed 1.5 years after sample collection and RNA extraction. During that time, the RNA eluents were stored at −80 °C and have been freeze-thawed multiple times, which can result in some loss of RNA [43]. It is also possible that the sample concentration and extraction steps applied for the passive samplers resulted in RNA fragmentation, which then prevented the RT and amplification of 3.9 kb of RNA sequences.

At the time of sampling, the UK was experiencing its third wave of the COVID-19 pandemic and the university had already implemented an infection mitigation plan to help protect the student population. This included attending classes online, non-pharmaceutical interventions, and the provision of an on-site testing facility. As cases of SARS-CoV-2 already existed in the student cohort at the commencement of sampling, it was not possible to critically evaluate its use as an early-warning system. Although the data collection here was undertaken with the full consent of the university, the information generated was not disseminated to the student cohort and was therefore of limited value in terms of changing student behaviour. This was partly due to a lack of knowledge about WBE and how to use the data effectively; however, this study provided the opportunity to put measures in place should another novel virus with pandemic potential emerge. It should be noted that a clinical testing centre for individuals was present on site and students were recommended to submit to regular testing to limit disease spread. Evidence from the on-site testing centre revealed that only a very small proportion of the students were being tested, suggesting testing fatigue among the cohort [44]. It is also worth noting that studies have noted a gender bias, with male students being less compliant [45, 46]. Overall, this highlights that WBE offers an unbiased way to assess the burden of disease in the complete student population when self-testing is failing. In addition, it has the added value of not placing undue stress on individuals when their levels of anxiety may also be heightened [42, 45].

This study clearly demonstrates that near-source sampling of wastewater can provide useful insights into the immediate health status of a population, especially when clinical and other surveillance approaches are limited. We found that this approach can indicate localized outbreaks, as shown with influenza A virus and enterovirus data. The early detection of such illnesses may be useful for the prevention of larger outbreaks and epidemics. We demonstrate that both qPCR and sequencing-based approaches are applicable for wastewater samples enriched using passive sampling; however, sample storage and RNA stability should be further explored. Furthermore, the use of passive samplers is a cost-effective and less labour-intensive approach to sampling and sample processing.

## Supporting information

Farkas et al. supplementary material Farkas et al. supplementary material

## Data Availability

Metadata are available in Supplementary Table S4.

## Supplementary material

For supplementary material accompanying this paper visit http://doi.org/10.1017/S0950268824000190. click here to view supplementary material

## References

[1] Rostami A, et al. (2021) SARS-CoV-2 seroprevalence worldwide: A systematic review and meta-analysis. Clinical Microbiology and Infection 27, 331–340. 33228974 10.1016/j.cmi.2020.10.020 PMC7584920

[2] Giacobbo A, et al. (2021) A critical review on SARS-CoV-2 infectivity in water and wastewater. What do we know? Science of the Total Environment 774, 145721. 33610994 10.1016/j.scitotenv.2021.145721 PMC7870439

[3] Fitzgerald SF, et al. (2021) Site specific relationships between COVID-19 cases and SARS-CoV-2 viral load in wastewater treatment plant influent. Environmental Science & Technology 55, 15276–15286. 34738785 10.1021/acs.est.1c05029

[4] Wade MJ, et al. (2022) Understanding and managing uncertainty and variability for wastewater monitoring beyond the pandemic: Lessons learned from the United Kingdom national COVID-19 surveillance programmes. Journal of Hazardous Materials 424, 127456. 34655869 10.1016/j.jhazmat.2021.127456 PMC8498793

[5] Ali W, et al. (2021) Occurrence of various viruses and recent evidence of SARS-CoV-2 in wastewater systems. Journal of Hazardous Materials 414, 125439. 33684818 10.1016/j.jhazmat.2021.125439 PMC7894103

[6] Nguyen AQ, et al. (2021) Monitoring antibiotic resistance genes in wastewater treatment: Current strategies and future challenges. Science of the Total Environment 783, 146964. 33866168 10.1016/j.scitotenv.2021.146964

[7] Sinclair RG, et al. (2008) Pathogen surveillance through monitoring of sewer systems. Advances in Applied Microbiology 65, 249–269. 19026868 10.1016/S0065-2164(08)00609-6 PMC7112011

[8] Zhang CM, et al. (2016) Elimination of viruses from domestic wastewater: Requirements and technologies. World Journal of Microbiology & Biotechnology 32, 1–9. 26931609 10.1007/s11274-016-2018-3

[9] Adriaenssens EM, et al. (2021) Tracing the fate of wastewater viruses reveals catchment-scale virome diversity and connectivity. Water Research 203, 117568. 34450465 10.1016/j.watres.2021.117568

[10] Graham KE, et al. (2021) SARS-CoV-2 RNA in wastewater settled solids is associated with COVID-19 cases in a large urban Sewershed. Environmental Science and Technology 55, 488–498. 33283515 10.1021/acs.est.0c06191

[11] Haak L, et al. (2022) Spatial and temporal variability and data bias in wastewater surveillance of SARS-CoV-2 in a sewer system. The Science of the Total Environment 805, 150390. 10.1016/J.SCITOTENV.2021.150390. 34818797 PMC8445773

[12] Nagarkar M, et al. (2022) SARS-CoV-2 monitoring at three sewersheds of different scales and complexity demonstrates distinctive relationships between wastewater measurements and COVID-19 case data. The Science of the Total Environment 816, 151534. 10.1016/J.SCITOTENV.2021.151534. 34780821 PMC8590472

[13] de Araújo JC, et al. (2023) Quantification of SARS-CoV-2 in wastewater samples from hospitals treating COVID-19 patients during the first wave of the pandemic in Brazil. Science of the Total Environment 860, 160498. 36436622 10.1016/j.scitotenv.2022.160498 PMC9691275

[14] Spurbeck RR, Minard-Smith A, Catlin L. (2021) Feasibility of neighborhood and building scale wastewater-based genomic epidemiology for pathogen surveillance. Science of the Total Environment 789, 147829. 34051492 10.1016/j.scitotenv.2021.147829 PMC8542657

[15] Shah S, et al. (2022) Wastewater surveillance to infer COVID-19 transmission: A systematic review. Science of the Total Environment 804, 150060. 34798721 10.1016/j.scitotenv.2021.150060 PMC8423771

[16] Schang C, et al. (2021) Passive sampling of SARS-CoV-2 for wastewater surveillance. Environmental Science and Technology 55, 10432–10441. 34264643 10.1021/acs.est.1c01530

[17] Blanco A, et al. (2019) Glass wool concentration optimization for the detection of enveloped and non-enveloped waterborne viruses. Food and Environmental Virology 11, 184–192. 30903596 10.1007/s12560-019-09378-0 PMC7090506

[18] Jones DL, et al. (2022) Critical evaluation of different passive sampler materials and approaches for the recovery of SARS-CoV-2, faecal-indicator viruses and bacteria from wastewater. Water 14, 3568.

[19] Corchis-Scott R, et al. (2021) Averting an outbreak of SARS-CoV-2 in a university residence hall through wastewater surveillance. Microbiology Spectrum 9 (2). 10.1128/spectrum.00792-21 PMC8510253 34612693

[20] Habtewold J, et al. (2022) Passive sampling, a practical method for wastewater-based surveillance of SARS-CoV-2. Environmental Research 204, 112058. 10.1016/j.envres.2021.112058 34516976 PMC8433097

[21] Kevill JL, et al. (2022) A comparison of precipitation and filtration-based SARS-CoV-2 recovery methods and the influence of temperature, turbidity, and surfactant load in urban wastewater. Science of the Total Environment 808, 151916. 34826466 10.1016/j.scitotenv.2021.151916 PMC8610557

[22] Farkas K, et al. (2022) Comparative assessment of filtration- and precipitation-based methods for the concentration of SARS-CoV-2 and other viruses from wastewater. Microbiology Spectrum 10 (4). 10.1128/SPECTRUM.01102-22 PMC9430619 35950856

[23] Farkas K, et al. (2023) Rapid assessment of SARS-CoV-2 variant-associated mutations in wastewater using real-time RT-PCR. Microbiology Spectrum 11 (1). 10.1128/spectrum.03177-22 PMC9927140 36629447

[24] Farkas K, et al. (2017) Evaluation of two triplex one-step qRT-PCR assays for the quantification of human enteric viruses in environmental samples. Food and Environmental Virology 9, 343–349. 10.1007/s12560-017-9293-5 PMC5548846 28391510

[25] Farkas K, et al. (2019) Critical evaluation of CrAssphage as a molecular marker for human-derived wastewater contamination in the aquatic environment. Food and Environmental Virology 11. 10.1007/s12560-019-09369-1 PMC6513805 30758724

[26] Child HT, et al. ( 2023) Optimised protocol for monitoring SARS-CoV-2 in wastewater using reverse complement PCR-based whole-genome sequencing. PLOS ONE 18, e0284211. 37058515 10.1371/journal.pone.0284211 PMC10104291

[27] Shaw AG, et al. (2020) Rapid and sensitive direct detection and identification of poliovirus from stool and environmental surveillance samples by use of nanopore sequencing. Journal of Clinical Microbiology 58 (9). 10.1128/JCM.00920-20/SUPPL_FILE/JCM.00920-20-SD002.XLSX PMC7448630 32611795

[28] Arita M, et al. (2015) Development of an efficient entire-capsid-coding-region amplification method for direct detection of poliovirus from stool extracts. Journal of Clinical Microbiology 53, 73–78. 25339406 10.1128/JCM.02384-14 PMC4290957

[29] Kilpatrick DR, et al. (2011) Poliovirus serotype-specific VP1 sequencing primers. Journal of Virological Methods 174, 128–130. 21440569 10.1016/j.jviromet.2011.03.020

[30] Karthikeyan S, et al. (2022) Wastewater sequencing reveals early cryptic SARS-CoV-2 variant transmission. Nature 609, 101–108. 35798029 10.1038/s41586-022-05049-6 PMC9433318

[31] COG-UK (2020) A nextflow pipeline for running the ARTIC network’s field bioinformatics tools. Available at https://github.com/artic-network/fieldbioinformatics, with a focus on ncov2019.

[32] Vincent-Hubert F, et al. (2021) Passive samplers, a powerful tool to detect viruses and Bacteria in marine coastal areas. Frontiers in Microbiology 12, 333. 10.3389/fmicb.2021.631174 PMC7940377 33708186

[33] Li J, et al. (2022) In situ calibration of passive samplers for viruses in wastewater. ACS EST Water 2 (11), 1881–1890. 10.1021/acsestwater.1c00406

[34] Bivins A, et al. (2022) Building-level wastewater surveillance using tampon swabs and RT-LAMP for rapid SARS-CoV-2 RNA detection. Environmental Science: Water Research and Technology 8, 173–183.

[35] Kevill JL, et al. (2022) Assessment of two types of passive sampler for the efficient recovery of SARS-CoV-2 and other viruses from wastewater. Science of the Total Environment 838, 156580. 10.1016/j.scitotenv.2022.156580 35690190 PMC9181630

[36] Vincent-Hubert F, et al. (2022) Development of passive samplers for the detection of SARS-CoV-2 in sewage and seawater: Application for the monitoring of sewage. Science of the Total Environment 833, 155139. 10.1016/j.scitotenv.2022.155139 35405243 PMC8993413

[37] Cinek O, et al. (2018) Quantitative CrAssphage real-time PCR assay derived from data of multiple geographically distant populations. Journal of Medical Virology 90, 767–771. 29297933 10.1002/jmv.25012

[38] Cha G, et al. (2023) Parallel deployment of passive and composite samplers for surveillance and variant profiling of SARS-CoV-2 in sewage. The Science of the Total Environment 866. 10.1016/J.SCITOTENV.2022.161101 PMC9792180 36581284

[39] Corchis-Scott R, et al. (2023) Actionable wastewater surveillance: Application to a university residence hall during the transition between Delta and omicron resurgences of COVID-19. Frontiers in Public Health 11, 1139423. 37265515 10.3389/fpubh.2023.1139423 PMC10230041

[40] Mejías-Molina C, et al. (2023) Effectiveness of passive sampling for the detection and genetic characterization of human viruses in wastewater. Environmental Science: Water Research & Technology 9, 1195–1204.

[41] PHE (2021) Weekly national Influenza and COVID-19 surveillance report Week 5 report (up to week 4 data).

[42] Heijnen L and Medema G. (2011) Surveillance of influenza A and the pandemic influenza A (H1N1) 2009 in sewage and surface water in the Netherlands. Journal of Water and Health 9, 434–442. 21976191 10.2166/wh.2011.019

[43] Thapar I, et al. (2023) Influence of storage conditions and multiple freeze-thaw cycles on N1 SARS-CoV-2, PMMoV, and BCoV signal. Science of the Total Environment 896, 165098. 37392884 10.1016/j.scitotenv.2023.165098 PMC10307669

[44] Mellou K, et al. (2022) Time lag between COVID-19 diagnosis and symptoms onset for different population groups: Evidence that self-testing in schools was associated with timely diagnosis among children. Life 12, 1305. 36143342 10.3390/life12091305 PMC9506207

[45] Berman AH, et al. (2022) Compliance with recommendations limiting COVID-19 contagion among university students in Sweden: Associations with self-reported symptoms, mental health and academic self-efficacy. Scandinavian Journal of Public Health 50, 70–84. 34213359 10.1177/14034948211027824 PMC8808007

[46] Green MA, et al. (2021) Evaluating social and spatial inequalities of large scale rapid lateral flow SARS-CoV-2 antigen testing in COVID-19 management: An observational study of Liverpool, UK (November 2020 to January 2021). The Lancet Regional Health – Europe 6, 100107. 34002172 10.1016/j.lanepe.2021.100107 PMC8114854

