# Supplementary material for: Near-source passive sampling for monitoring viral outbreaks within a university residential setting
Source: Epidemiol Infect. 2024 Feb 8;152:e31. doi: 10.1017/S0950268824000190 (PMC10894896; doi:10.1017/S0950268824000190)
Supplement: Farkas et al. supplementary material [file S0950268824000190sup001.docx]

Epidemiology and Infection

Near-source passive sampling for monitoring viral outbreaks within a university residential setting

Supplementary material

Kata Farkas^1†*^, Jessica L. Kevill^1†^, Latifah Adwan^1^, Alvaro Garcia-Delgado^1^, Rande Dzay^1^, Jasmine M.S. Grimsley^2,3^, Kathryn Lambert-Slosarska^1^, Matthew J. Wade^2,4^, Rachel C. Williams^1^, Javier Martin^5^, Mark Drakesmith^6^, Jiao Song^6^, Victoria McClure^6^, Davey L. Jones^1,7^

^1^ School of Environmental and Natural Sciences, Bangor University, Bangor, Gwynedd LL57 2UW, UK

^2^ Data Analytics & Surveillance Group, UK Health Security Agency, 10 South Colonnade, Canary Wharf, London E14 4PU, UK

^3^ The London Data Company, London EC2N 2AT, UK

^4^ School of Engineering, Newcastle University, Newcastle-upon-Tyne NE1 7RU, UK

^5^ Division of Vaccines, Medicines and Healthcare products Regulatory Agency, Potters Bar, Hertfordshire EN6 3QG, UK

^6^ Communicable Disease Surveillance Centre, Public Health Wales, 2 Capital Quarter, Tyndall Street, Cardiff, CF10 4BZ, UK

^7^ Food Futures Institute, Murdoch University, 90 South Street, Murdoch, WA 6150, Australia

^†^Joint first author

* Corresponding author:

Kata Farkas, PhD

[fkata211@gmail.com](mailto:fkata211@gmail.com)


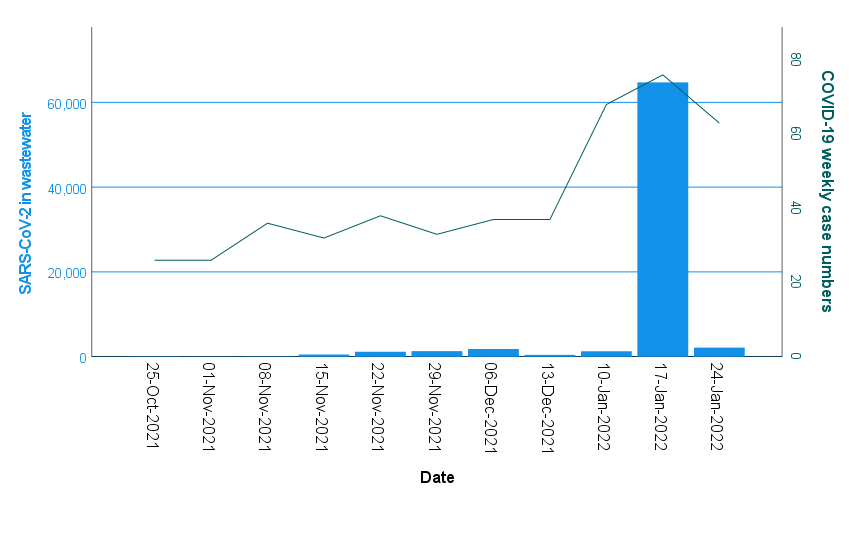


**Figure S1** Cumulative SARS-CoV-2 concentrations (gc/sampler; bars) measured at Sites 1-4 and the weekly COVID-19 case numbers at Bangor Hospital (line) during the study period. The case data refers to patients who were inpatients in hospital at the end of each week and had tested positive for COVID-19 in the previous 28 days. The numbers do not include hospital visits where the patient was not admitted. (Case numbers were kindly provided by Public Health Wales.)


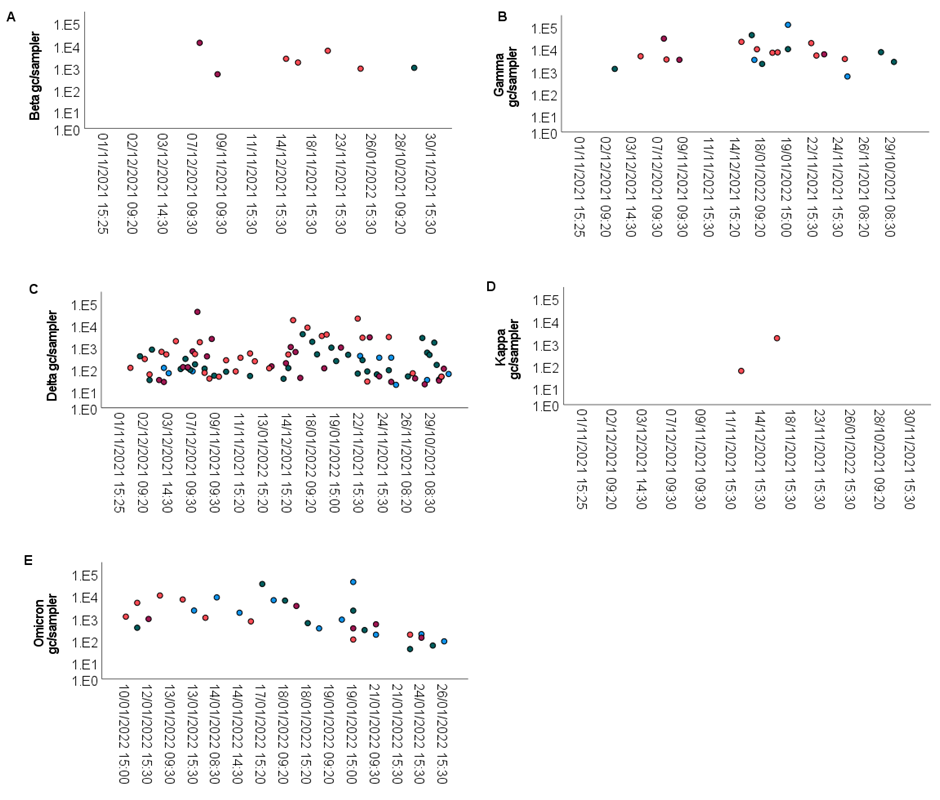
**Figure S2** Concentrations of SARS-CoV-2 sequences with mutations associated with A) Beta, B) Gamma, C) Delta, D) Kappa and E) Omicron variants in passive samplers deployed in the sewer network in a university residential setting at Site 1; Ffriddoedd West residential block (blue), Site 2; Ffriddoedd East residential block (green), Site 3; Sports facility – Brailsford (purple) and Site 4; St Mary’s residential block (red).

**Table S1.** Primers and probes used withing in this study.

| Target | Target mutation/region | Reference | Oligo  type | Sequence (5'-3') | Ta | Target DNA sequence (5'-3') |
| --- | --- | --- | --- | --- | --- | --- |
| SARS-CoV-2 | N1 | (1) | Forward | GACCCCAAAATCAGCGAAAT | 60 | gtgaaatggtcatgtgtggcggttcactatatgttaaaccaggtggaacctcatcaggagatgccacaactgcttatgctaatagtgtttttaaca  tttg |
|  |  |  | Reverse | TCTGGTTACTGCCAGTTGAATCTG |  |  |
|  |  |  | Probe | FAM-ACCCCGCATTACGTTTGGTGGACC-MGB |  |  |
| Phi6 | N gene | (2) | Forward | TGGCGGCGGTCAAGAGC | 60 | TGGCGGCGGTCAAGAGCAACCCGGTCGTCGCAGGTCTGACACTCGCTCAGATCGGAAGCACCGGTTATGACGCCTATCAGCAGCTTCTGGAGAATCATCC |
|  |  |  | Reverse | GGATGATTCTCCAGAAGCTGCTG |  |  |
|  |  |  | Probe | VIC-CGGTCGTCGCAGGTCTGACACTCGC -QSY |  |  |
| SARS-CoV-2 Beta | K417N | TBC | Forward | TGAAGTCAGACAAATCGCTCC | 58 | TGATGAAGTCAGACAAATCGCTCCAGGGCAAACTGGAAATATTGCTGATTATAATTATAAATTACCAGATGATTTTACAGGCTGCGTTATAGCTTGGAATTCTAA |
|  |  |  | Reverse | CAAGCTATAACGCAGCCTGT |  |  |
|  |  |  | Probe | HEX-AGGGCAAACTGGAAATATTGCTG-BHQ |  |  |
| SARS-CoV-2 Gamma | K417T | TBC | Forward | TGAAGTCAGACAAATCGCTCC | 58 | TGATGAAGTCAGACAAATCGCTCCAGGGCAAACTGGAACGATTGCTGATTATAATTATAAATTACCAGATGATTTTACAGGCTGCGTTATAGCTTGGAATTCTAA |
|  |  |  | Reverse | CAAGCTATAACGCAGCCTGT |  |  |
|  |  |  | Probe | FAM-ACTGGAACGATTGCTGATTATAATT-MGB |  |  |
| SARS-CoV-2 Kappa | E154K | TBC | Forward | GCCGGTAGCACACCTTGTAA | 58 | CAACTGAAATCTATCAGGCCGGTAGCACACCTTGTAATGGTGTTCAAGGTTTTAATTGTTACTTTCCTTTACAATCATATGGTTTCCAACCCACTAATGGTG |
|  |  |  | Reverse | GTTGGAAACCATATGATTGTAAAGGA |  |  |
|  |  |  | Probe | HEX-TGGTGTTCAAGGTTTTAATTGTTAC-BHQ |  |  |
| SARS-CoV-2 Delta | DEL156-157 | TBC | Forward | GATCCATTTTTGGGTGTTTATTACC | 58 | GATCCATTTTTGGGTGTTTATTACCACAAAAACAACAAAAGTTGGATGGAAAGTAGAGTTTATTCTAGTGCGAATAATTGCACTTTTGAATATGTCTCTCAGCC |
|  |  |  | Reverse | GGCTGAGAGACATATTCAAAAGTG |  |  |
|  |  |  | Probe | FAM-TGGAAAGTAGAGTTTATTCTAGTGCG-MGB |  |  |
| SARS-CoV-2 Omicron | DEL156-157 | This study | Forward | GTGTTGCTGATTATTCTGTCC | 58 | GTGTTGCTGATTATTCTGTCCTATATAAT(CorT)TCGCACCATTTTTCACTTTTAAGTGTTATGGAGTGTCTCCTACTAAATTAAATGATCTCTGCTTTACTAATGTCTATGC |
|  |  |  | Reverse | GCATAGACATTAGTAAAGCAGAG |  |  |
|  |  |  | Probe | HEX-YTCGCACCATTTTTCRCTTTTAAGTG-BHQ |  |  |
| Influenza A | Segment 7 matrix protein 2 gene | (3) | Forward | CAAGACCAATCYTGTCACCTCTGAC | 60 | AAAGACAAGACCAATCCTGTCACCTCTGACTAAGGGGATTTTAGGATTTGTGTTCACGCTCACCGTGCCCAGTGAGCGAGGACTGCAGCGTAGACGCTTTGTCCAAAATGCCCTAAATGGG |
|  |  |  |  | CAAGACCAATYCTGTCACCTYTGAC |  |  |
|  |  |  | Reverse | GCATTYTGGACAAAVCGTCTACG |  |  |
|  |  |  |  | GCATTTTGGATAAAGCGTCTACG |  |  |
|  |  |  | Probe | FAM-TGCAGTCCT-ZEN-CGCTCACTGGGCACG-IABkFQ |  |  |
| Influenza B | Segment 8 nonstructural protein 1 | (3) | Forward | TCCTCAAYTCACTCTTCGAGCG | 60 | GGATCCTCAACTCACTCTTCGAGCGTTTTGATGAAGGACATTCAAAGCCAATTCGAGCAGCTGAAACTGCGGTGGGAGTCTTATCCCAATTTGGTCAAGAGCACCGATT |
|  |  |  | Reverse | CGGTGCTCTTGACCAAATTGG |  |  |
|  |  |  | Probe | YakYel-CCAATTCGA-ZEN-GCAGCTGAAACTGCGGTG-IABkFQ |  |  |
| Enteroviruses (EV) | 5′UTR | Public  Health  Wales | Forward | GGTYGAAGAGYCTATTGAGC | 60 | ATATGGACATGGTGCAAAGAGTCTATTGAGCTAGTTAGTAGTCCTCCGGCCCCTGAATGCGGCTAATCCTAACTGCGGAGCACATACCCTC |
|  |  |  | Reverse | GCTCCGYIGTTRGGATTAGCCG |  |  |
|  |  |  | Probe | HEX-TCCTCCGGCCCCTG-BHQ |  |  |
| Enterovirus D68 (EV-D68) | 5′UTR | (4) | Forward | TGTTCCCACGGTTGAAAACAA | 60 | TTGTATAAACTGTTCCCACGGTTGAAAACAACCTATCCGTTAT/ACCGCTATAGTACTTCGAGAAACCTAGTATCACCTTTGGATTGTTGACGCGTTGCGCTCAGCACACTAACC  CGTGTGTAGCTTGGGTCGATGAGTCTGGACATACCCCACTGGCGACAGTGGTCCAGGCTGCGTTGGCGGCCTACTCATGGTGAAAACCATGAGACGCTAGACATGAACAAGGT |
|  |  |  | Reverse | TGTCTAGCGTCTCATGGTTTTCAC |  |  |
|  |  |  | Probe | FAM-TCCGCTATAGTACTTCG-MGB |  |  |
|  |  |  | Probe | FAM-ACCGCTATAGTACTTCG-MGB |  |  |
| CrAssphage | single stranded DNA-binding protein gene | (5) | Forward | CAGAAGTACAAACTCCTAAAAAACGTAGAG | 60 | CAGAAGTACAAACTCCTAAAAAACGTAGAGGTAGAGGTATTAATAACGATTTACGTGATGTAACTCGTAAAAAGTTTGATGAACGTACTGATTGTAATAAAGCTAATGGCTTGTTTATTGGTC |
|  |  |  | Reverse | GATGACCAATAAACAAGCCATTAGC |  |  |
|  |  |  | Probe | FAM-AATAACGATTTACGTGATGTAAC-TAMRA |  |  |

| Viral target | Genome | Kit | Cycling conditions |
| --- | --- | --- | --- |
| SARS-CoV-2 (All targets) | RNA | TaqMan Fast Virus 1-Step Master Mix | 50^o^C 30 min, 95^o^C 20 sec, x 45 cycles of 95^o^C 0.03 sec, Ta (see Table S1) 45 s |
| Phi6 |  |  |  |
| Influenza-A |  |  |  |
| Influenza-B |  |  |  |
| Enterovirus |  |  |  |
| D68 |  |  |  |
| CrAssphage | DNA | QuantiNova Probe PCR Kit | 95°C 2 min, x 40 cycles of 95^o^C 15 sec, 60°C 1 min |

**Table S2.** qPCR cycling conditions used in the analysis of the target viruses.

**Table S3.** Spearman (2-tailed) correlation between crAssphage, SARS-CoV-2 (N1 gene), influenza A virus, *Enterovirus* spp. and enterovirus D68.

|  |  |  | CrAssphage | SARS-CoV-2 | | Influenza A virus | *Enterovirus* spp. | Enterovirus D68 |
| --- | --- | --- | --- | --- | --- | --- | --- | --- |
| CrAssphage | rho | | 1.000 | | -0.139^*^ | 0.523^**^ | 0.112 | 0.064 |
|  | p | | . | | 0.017 | <0.001 | 0.056 | 0.278 |
|  | n | | 295 | | 295 | 294 | 293 | 293 |
| SARS-CoV-2 | rho | | -0.139^*^ | | 1.000 | -0.030 | 0.035 | -0.008 |
|  | p | | 0.017 | | . | 0.607 | 0.555 | 0.888 |
|  | n | | 295 | | 295 | 294 | 293 | 293 |
| Influenza A virus | rho | | 0.523^**^ | | -0.030 | 1.000 | -0.016 | -0.036 |
|  | p | | <0.001 | | 0.607 | . | 0.780 | 0.536 |
|  | n | | 294 | | 294 | 294 | 293 | 293 |
| *Enterovirus* spp. | rho | | 0.112 | | 0.035 | -0.016 | 1.000 | 0.351^**^ |
|  | p | | 0.056 | | 0.555 | 0.780 | . | <0.001 |
|  | n | | 293 | | 293 | 293 | 293 | 293 |
| Enterovirus D68 | rho | | 0.064 | | -0.008 | -0.036 | 0.351^**^ | 1.000 |
|  | p | | 0.278 | | 0.888 | 0.536 | <0.001 | . |
|  | n | | 293 | | 293 | 293 | 293 | 293 |
|  | p | | 0.126 | | 0.164 | 0.601 | . | . |
|  | n | | 74 | | 74 | 74 | 74 | 74 |

**Table S4.** Metadata on the viral concentrations in each sample collected.

| Site Name | Sample time | Comp/passive start time | comp passive end time | Phi Rec% | Phi Rec% RR | CrAss (gc/l) | SAR-CoV (gc/l) | EV (gc / l) | EV-D68 (gc / l) | NoVGII (gc / l) | Flu-A (gc / l) | Flu-B (gc / l) | Beta (gc / l) | Delta (gc / l) | Gamma (gc / l) | Kappa (gc / l) | Omicron (gc / l) |
| --- | --- | --- | --- | --- | --- | --- | --- | --- | --- | --- | --- | --- | --- | --- | --- | --- | --- |
| St Mary's | 25/10/2021 15:30 | 25/10/2021 09:15 | 25/10/2021 15:30 | 0.0000 | 0.0000 | 6184406 | 181 | 0 | 0 |  | 0 | 0 | 0 | 0 | 0 | 0 |  |
| Ffriddoedd site-Road | 25/10/2021 15:30 | 25/10/2021 09:25 | 25/10/2021 15:30 | 0.0000 | 0.0000 | 87076 | 0 | 0 | 0 |  | 0 | 0 | 0 | 0 | 0 | 0 |  |
| Ffriddoedd site-Brailsford | 25/10/2021 15:30 | 25/10/2021 09:35 | 25/10/2021 15:30 | 0.0875 | 0.0000 | 7092897 | 0 | 0 | 0 |  | 0 | 0 | 0 | 0 | 0 | 0 |  |
| Ffriddoedd site-Reichel | 25/10/2021 15:30 | 25/10/2021 09:40 | 25/10/2021 15:30 | 0.0000 | 0.0000 | 6240321 | 0 | 0 | 0 |  | 0 | 0 | 0 | 0 | 0 | 0 |  |
| St Mary's | 26/10/2021 09:00 | 25/10/2021 15:30 | 26/10/2021 09:00 | 0.0000 | 0.0000 | 14338732 | 0 | 0 | 0 |  | 0 | 0 | 0 | 0 | 0 | 0 |  |
| Ffriddoedd site-Road | 26/10/2021 09:00 | 25/10/2021 15:30 | 26/10/2021 09:00 | 0.0000 | 0.0000 | 13450617 | 0 | 0 | 0 |  | 0 | 0 | 0 | 0 | 0 | 0 |  |
| Ffriddoedd site-Brailsford | 26/10/2021 09:00 | 25/10/2021 15:30 | 26/10/2021 09:00 | 0.0000 | 0.0000 | 7435197 | 0 | 0 | 0 |  | 0 | 0 | 0 | 0 | 0 | 0 |  |
| Ffriddoedd site-Reichel | 26/10/2021 09:00 | 25/10/2021 15:30 | 26/10/2021 09:00 | 0.0000 | 0.0000 | 1930224 | 0 | 0 | 0 |  | 0 | 0 | 0 | 0 | 0 | 0 |  |
| St Mary's | 27/10/2021 09:00 | 26/10/2021 15:30 | 27/10/2021 09:00 | 0.0000 | 0.0000 | 0 | 0 | 0 | 0 |  | 0 | 0 | 0 | 0 | 0 | 0 |  |
| Ffriddoedd site-Road | 27/10/2021 09:00 | 26/10/2021 15:30 | 27/10/2021 09:00 | 0.4720 | 0.4193 | 3512736 | 0 | 0 | 0 |  | 0 | 0 | 0 | 0 | 0 | 0 |  |
| Ffriddoedd site-Brailsford | 27/10/2021 09:00 | 26/10/2021 15:30 | 27/10/2021 09:00 | 0.0000 | 0.0000 | 0 | 0 | 0 | 0 |  | 0 | 0 | 0 | 0 | 0 | 0 |  |
| Ffriddoedd site-Reichel | 27/10/2021 09:00 | 26/10/2021 15:30 | 27/10/2021 09:00 | 0.0000 | 0.0000 | 5576932 | 0 | 0 | 0 |  | 0 | 0 | 0 | 0 | 0 | 0 |  |
| St Mary's | 28/10/2021 09:00 | 27/10/2021 15:30 | 28/10/2021 09:00 | 0.2761 | 0.2515 | 7763890 | 8 | 0 | 0 |  | 0 | 0 | 0 | 0 | 0 | 0 |  |
| Ffriddoedd site-Road | 28/10/2021 09:10 | 27/10/2021 15:30 | 28/10/2021 09:00 | 0.2707 | 0.3911 | 1197004 | 55 | 0 | 82.27 |  | 0 | 0 | 0 | 21.84 | 0 | 0 |  |
| Ffriddoedd site-Brailsford | 28/10/2021 09:15 | 27/10/2021 15:30 | 28/10/2021 09:00 | 0.2888 | 0.2383 | 19385093 | 19411 | 0 | 140.84 |  | 0 | 0 | 0 | 2756.47 | 7409.47 | 0 |  |
| Ffriddoedd site-Reichel | 28/10/2021 09:20 | 27/10/2021 15:30 | 28/10/2021 09:00 | 0.0995 | 0.0537 | 9485193 | 122 | 0 | 0 |  | 0 | 0 | 0 | 0 | 0 | 0 |  |
| St Mary's | 29/10/2021 08:05 | 28/10/2021 15:30 | 29/10/2021 09:00 | 45.3596 |  | 0 | 0 | 0 | 0 |  | 0 | 0 | 0 | 0 | 0 | 0 |  |
| Ffriddoedd site-Road | 29/10/2021 08:15 | 28/10/2021 15:30 | 29/10/2021 09:00 | 0.0652 | 0.0042 | 13718278 | 624 | 0 | 0 |  | 0 | 0 | 0 | 0 | 0 | 0 |  |
| Ffriddoedd site-Brailsford | 29/10/2021 08:25 | 28/10/2021 15:30 | 29/10/2021 09:00 | 0.1637 |  | 64315135 | 2585 | 0 | 0 |  | 0 | 0 | 0 | 478.16 | 0 | 0 |  |
| Ffriddoedd site-Reichel | 29/10/2021 08:30 | 28/10/2021 15:30 | 29/10/2021 09:00 | 0.1665 |  | 5356096 | 0 | 994969.5 | 992708.9 |  | 0 | 0 | 0 | 0 | 0 | 0 |  |
| St Mary's | 26/10/2021 15:30 | 26/10/2021 09:00 | 26/10/2021 15:30 | 0.0000 | 0.0000 | 0 | 277 | 0 | 0 |  | 0 | 0 | 0 | 0 | 0 | 0 |  |
| Ffriddoedd site-Road | 26/10/2021 15:30 | 26/10/2021 09:00 | 26/10/2021 15:30 | 0.6706 | 0.9226 | 10970992 | 0 | 0 | 0 |  | 0 | 0 | 0 | 0 | 0 | 0 |  |
| Ffriddoedd site-Brailsford | 26/10/2021 15:30 | 26/10/2021 09:00 | 26/10/2021 15:30 | 0.0000 | 0.0000 | 0 | 0 | 0 | 0 |  | 0 | 0 | 0 | 0 | 0 | 0 |  |
| Ffriddoedd site-Reichel | 26/10/2021 15:30 | 26/10/2021 09:00 | 26/10/2021 15:30 | 0.0000 | 0.0000 | 7402834 | 0 | 0 | 0 |  | 0 | 0 | 0 | 0 | 0 | 0 |  |
| St Mary's | 27/10/2021 15:30 | 27/10/2021 09:00 | 27/10/2021 15:30 | 0.0234 | 0.0013 | 11493520 | 0 | 0 | 0 |  | 0 | 0 | 0 | 0 | 0 | 0 |  |
| Ffriddoedd site-Road | 27/10/2021 15:30 | 27/10/2021 09:00 | 27/10/2021 15:30 | 0.1638 | 0.3376 | 1367844 | 46 | 0 | 0 |  | 0 | 0 | 0 | 0 | 0 | 0 |  |
| Ffriddoedd site-Brailsford | 27/10/2021 15:30 | 27/10/2021 09:00 | 27/10/2021 15:30 | 0.0000 | 0.1427 | 21188099 | 44 | 0 | 0 |  | 0 | 0 | 0 | 0 | 0 | 0 |  |
| Ffriddoedd site-Reichel | 27/10/2021 15:30 | 27/10/2021 09:00 | 27/10/2021 15:30 | 0.0000 | 0.0008 | 16630316 | 0 | 0 | 633.53 |  | 0 | 0 | 0 | 0 | 0 | 0 |  |
| St Mary's | 28/10/2021 15:30 | 28/10/2021 09:00 | 28/10/2021 15:30 | 0.1123 |  | 16929027 | 0 | 0 | 0 |  | 0 | 0 | 0 | 0 | 0 | 0 |  |
| Ffriddoedd site-Road | 28/10/2021 15:30 | 28/10/2021 09:00 | 28/10/2021 15:30 | 0.2210 |  | 5465748 | 0 | 0 | 0 |  | 0 | 0 | 0 | 0 | 0 | 0 |  |
| Ffriddoedd site-Brailsford | 28/10/2021 15:30 | 28/10/2021 09:00 | 28/10/2021 15:30 | 0.0441 | 0.1420 | 4956133 | 21357 | 0 | 0 |  | 0 | 0 | 0 | 602.34 | 0 | 0 |  |
| Ffriddoedd site-Reichel | 28/10/2021 15:30 | 28/10/2021 09:00 | 28/10/2021 15:30 | 0.5288 |  | 94355263 | 132 | 0 | 33.36 |  | 0 | 0 | 0 | 34.66 | 0 | 0 |  |
| St Mary's | 29/10/2021 15:30 | 29/10/2021 09:00 | 29/10/2021 15:30 | 0.2251 |  | 13004784 | 0 | 0 | 0 |  | 0 | 0 | 0 | 0 | 0 | 0 |  |
| Ffriddoedd site-Road | 29/10/2021 15:30 | 29/10/2021 09:00 | 29/10/2021 15:30 | 1.2450 |  | 5621819 | 0 | 1518.99 | 67.8 |  | 7.58 | 0 | 0 | 0 | 0 | 0 |  |
| Ffriddoedd site-Brailsford | 29/10/2021 15:30 | 29/10/2021 09:00 | 29/10/2021 15:30 | 0.2000 |  | 12393108 | 13090 | 0 | 283.01 |  | 0 | 0 | 1027.23 | 1707.44 | 2717.75 | 0 |  |
| Ffriddoedd site-Reichel | 29/10/2021 15:30 | 29/10/2021 09:00 | 29/10/2021 15:30 | 0.1147 |  | 25902520 | 0 | 0 | 484.34 |  | 0 | 0 | 0 | 0 | 0 | 0 |  |
| St Mary's | 01/11/2021 15:00 | 01/11/2021 09:00 | 01/11/2021 15:00 | 0.0291 | 0.0391 | 26336266 | 0 | 0 | 0 |  | 13.11 | 0 | 0 | 0 | 0 | 0 |  |
| Ffriddoedd site-Road | 01/11/2021 15:10 | 01/11/2021 09:10 | 01/11/2021 15:10 | 11.6487 |  | 6805608 | 0 | 0 | 0 |  | 0 | 0 | 0 | 0 | 0 | 0 |  |
| Ffriddoedd site-Brailsford | 01/11/2021 15:25 | 01/11/2021 09:25 | 01/11/2021 15:25 | 0.0646 | 0.1119 | 54355136 | 0 | 0 | 0 |  | 0 | 0 | 0 | 0 | 0 | 0 |  |
| Ffriddoedd site-Reichel | 01/11/2021 15:35 | 01/11/2021 09:35 | 01/11/2021 15:35 | 0.1323 |  | 13969569 | 52 | 0 | 0 |  | 0 | 0 | 0 | 0 | 0 | 0 |  |
| St Mary's | 02/11/2021 09:00 | 01/11/2021 15:00 | 02/11/2021 15:00 | 0.0166 | 0.0262 | 22831623 | 0 | 0 | 0 |  | 0 | 0 | 0 | 0 | 0 | 0 |  |
| Ffriddoedd site-Road | 02/11/2021 09:10 | 01/11/2021 15:10 | 02/11/2021 15:10 | 0.3075 |  | 35511898 | 0 | 0 | 0 |  | 0 | 0 | 0 | 0 | 0 | 0 |  |
| Ffriddoedd site-Brailsford | 02/11/2021 09:25 | 01/11/2021 15:25 | 02/11/2021 15:25 | 0.1609 |  | 36451934 | 73 | 0 | 0 |  | 0 | 0 | 0 | 2.74 | 0 | 0 |  |
| Ffriddoedd site-Reichel | 02/11/2021 09:35 | 01/11/2021 15:35 | 02/11/2021 15:35 | 0.0537 | 0.0057 | 42830903 | 77 | 0 | 0 |  | 0 | 0 | 0 | 0 | 0 | 0 |  |
| St Mary's | 02/11/2021 15:00 | 02/11/2021 09:00 | 02/11/2021 15:00 | 0.0000 | 0.0000 | 28180288 | 0 | 0 | 0 |  | 0 | 0 | 0 | 0 | 0 | 0 |  |
| Ffriddoedd site-Road | 02/11/2021 15:10 | 02/11/2021 09:10 | 02/11/2021 15:10 | 0.0000 | 0.0000 | 8295479 | 0 | 0 | 0 |  | 0 | 0 | 0 | 0 | 0 | 0 |  |
| Ffriddoedd site-Brailsford | 02/11/2021 15:25 | 02/11/2021 09:25 | 02/11/2021 15:25 | 0.0000 | 0.0000 | 49618578 | 5117 | 1631.27 | 0 |  | 0 | 0 | 0 | 415.3 | 0 | 0 |  |
| Ffriddoedd site-Reichel | 02/11/2021 15:35 | 02/11/2021 09:35 | 02/11/2021 15:35 | 0.0000 | 0.0000 | 3731027 | 0 | 0 | 0 |  | 0 | 0 | 0 | 0 | 0 | 0 |  |
| St Mary's | 03/11/2021 09:00 | 02/11/2021 15:00 | 03/11/2021 09:00 | 0.0000 | 0.0000 | 25604874 | 0 | 0 | 0 |  | 0 | 0 | 0 | 0 | 0 | 0 |  |
| Ffriddoedd site-Road | 03/11/2021 09:10 | 02/11/2021 15:10 | 03/11/2021 09:10 | 0.0000 | 0.0000 | 7222987 | 0 | 0 | 0 |  | 0 | 0 | 0 | 0 | 0 | 0 |  |
| Ffriddoedd site-Brailsford | 03/11/2021 09:25 | 02/11/2021 15:25 | 03/11/2021 09:25 | 0.0000 | 0.0000 | 34974175 | 5712 | 0 | 0 |  | 0 | 0 | 0 | 825.49 | 1324.02 | 0 |  |
| Ffriddoedd site-Reichel | 03/11/2021 09:35 | 02/11/2021 15:35 | 03/11/2021 09:35 | 0.0000 | 0.0000 | 6784372 | 0 | 0 | 0 |  | 0 | 0 | 0 | 0 | 0 | 0 |  |
| St Mary's | 03/11/2021 15:00 | 03/11/2021 09:00 | 03/11/2021 15:00 | 0.6660 |  | 35355366 | 0 | 0 | 0 |  | 0 | 0 | 0 | 0 | 0 | 0 |  |
| Ffriddoedd site-Road | 03/11/2021 15:10 | 03/11/2021 09:10 | 03/11/2021 15:10 | 0.7428 |  | 3560698 | 71 | 0 | 0 |  | 0 | 0 | 0 | 34.43 | 0 | 0 |  |
| Ffriddoedd site-Brailsford | 03/11/2021 15:20 | 03/11/2021 09:20 | 03/11/2021 15:20 | 0.4945 |  | 10376216 | 529 | 295.03 | 0 |  | 0 | 0 | 0 | 0 | 0 | 0 |  |
| Ffriddoedd site-Reichel | 03/11/2021 15:30 | 03/11/2021 09:30 | 03/11/2021 15:30 | 0.4212 |  | 44413959 | 0 | 0 | 0 |  | 0 | 0 | 0 | 0 | 0 | 0 |  |
| St Mary's | 04/11/2021 09:00 | 03/11/2021 15:00 | 04/11/2021 09:00 | 0.4002 |  | 9359961 | 0 | 0 | 0 |  | 0 | 0 | 0 | 0 | 0 | 0 |  |
| Ffriddoedd site-Road | 04/11/2021 09:10 | 03/11/2021 15:10 | 04/11/2021 09:10 | 0.8196 |  | 6114483 | 23 | 0 | 0 |  | 0 | 0 | 0 | 14.16 | 0 | 0 |  |
| Ffriddoedd site-Brailsford | 04/11/2021 09:20 | 03/11/2021 15:20 | 04/11/2021 09:20 | 0.1048 |  | 14264004 | 0 | 2391.65 | 0 |  | 0 | 0 | 0 | 0 | 0 | 0 |  |
| Ffriddoedd site-Reichel | 04/11/2021 09:30 | 03/11/2021 15:30 | 04/11/2021 09:30 | 0.5508 |  | 19906260 | 0 | 4738.98 | 0 |  | 0 | 0 | 0 | 0 | 0 | 0 |  |
| St Mary's | 04/11/2021 15:00 | 04/11/2021 09:00 | 04/11/2021 15:00 | 0.7588 |  | 20456647 | 0 | 0 | 0 |  | 0 | 0 | 0 | 0 | 0 | 0 |  |
| Ffriddoedd site-Road | 04/11/2021 15:10 | 04/11/2021 09:10 | 04/11/2021 15:10 | 1.0757 |  | 11637611 | 0 | 0 | 0 |  | 0 | 0 | 0 | 0 | 0 | 0 |  |
| Ffriddoedd site-Brailsford | 04/11/2021 15:20 | 04/11/2021 09:20 | 04/11/2021 15:20 | 1.9721 |  | 17936396 | 0 | 1732.79 | 41.7 |  | 0 | 0 | 0 | 0 | 0 | 0 |  |
| Ffriddoedd site-Reichel | 04/11/2021 15:30 | 04/11/2021 09:30 | 04/11/2021 15:30 | 0.2293 |  | 4678219 | 0 | 238 | 0 |  | 0 | 0 | 0 | 0 | 0 | 0 |  |
| St Mary's | 05/11/2021 09:00 | 04/11/2021 15:00 | 05/11/2021 09:00 | 2.6130 |  | 393470 | 7515 | 0 | 0 |  | 0 | 0 | 0 | 1985.67 | 4883.37 | 0 |  |
| Ffriddoedd site-Road | 05/11/2021 09:10 | 04/11/2021 15:10 | 05/11/2021 09:10 | 0.9556 |  | 1523758 | 226 | 0 | 0 |  | 0 | 0 | 0 | 133.91 | 0 | 0 |  |
| Ffriddoedd site-Brailsford | 05/11/2021 09:20 | 04/11/2021 15:10 | 05/11/2021 09:20 | 2.0059 |  | 11205912 | 307 | 1878.03 | 219.77 |  | 0 | 0 | 0 | 109.7 | 0 | 0 |  |
| Ffriddoedd site-Reichel | 05/11/2021 09:30 | 04/11/2021 15:10 | 05/11/2021 09:30 | 0.2485 |  | 23443374 | 0 |  |  |  | 0 | 0 | 0 | 0 | 0 | 0 |  |
| St Mary's | 09/11/2021 09:00 | 08/11/2021 15:00 | 09/11/2021 09:00 | 0.3122 |  | 159247980 | 35598 | 2473.32 | 0 |  | 0 | 0 | 0 | 1779.02 | 3485.41 | 0 |  |
| Ffriddoedd site-Road | 09/11/2021 09:10 | 08/11/2021 15:10 | 09/11/2021 09:10 | 0.0190 |  | 2883379 | 2 | 0 | 0 |  | 35983.71 | 0 | 0 | 0 | 0 | 0 |  |
| Ffriddoedd site-Brailsford | 09/11/2021 09:20 | 08/11/2021 15:10 | 09/11/2021 09:20 | 0.0170 | 0.0296 | 3910218 | 613 | 1138.09 | 0 |  | 0 | 0 | 0 | 54.55 | 0 | 0 |  |
| Ffriddoedd site-Reichel | 09/11/2021 09:30 | 08/11/2021 15:10 | 09/11/2021 09:30 | 0.0471 |  | 17949120 | 0 | 0 | 0 |  | 0 | 0 | 0 | 0 | 0 | 0 |  |
| St Mary's | 09/11/2021 15:00 | 09/11/2021 09:00 | 09/11/2021 15:00 | 0.1582 |  | 63706647 | 103 | 0 | 0 |  | 0 | 0 | 0 | 0 | 0 | 0 |  |
| Ffriddoedd site-Road | 09/11/2021 15:10 | 09/11/2021 09:10 | 09/11/2021 15:10 | 0.1965 |  | 8630216 | 0 | 0 | 0 |  | 0 | 0 | 0 | 0 | 0 | 0 |  |
| Ffriddoedd site-Brailsford | 09/11/2021 15:20 | 09/11/2021 09:20 | 09/11/2021 15:20 | 0.3166 |  | 52449300 | 0 | 506.59 | 0 |  | 0 | 0 | 0 | 0 | 0 | 0 |  |
| Ffriddoedd site-Reichel | 09/11/2021 15:30 | 09/11/2021 09:30 | 09/11/2021 15:30 | 0.0058 | 0.0000 | 5588277 | 0 | 0 | 0 |  | 0 | 0 | 0 | 0 | 0 | 0 |  |
| St Mary's | 10/11/2021 09:00 | 09/11/2021 15:00 | 10/11/2021 09:00 | 0.0191 | 0.0137 | 6571974 | 0 | 588.05 | 56.5 |  | 0 | 0 | 0 | 49.31 | 0 | 0 |  |
| Ffriddoedd site-Road | 10/11/2021 09:10 | 09/11/2021 15:10 | 10/11/2021 09:10 | 0.2604 |  | 7759724 | 0 | 0 | 0 |  | 40775.14 | 0 | 0 | 0 | 0 | 0 |  |
| Ffriddoedd site-Brailsford | 10/11/2021 09:20 | 09/11/2021 15:10 | 10/11/2021 09:20 | 0.0224 | 0.0043 | 9449810 | 1954 | 4446.15 | 0 |  | 0 | 0 | 0 | 82.84 | 0 | 0 |  |
| Ffriddoedd site-Reichel | 10/11/2021 09:30 | 09/11/2021 15:10 | 10/11/2021 09:30 | 0.1307 |  | 37121230 | 0 | 0 | 0 |  | 0 | 0 | 0 | 0 | 0 | 0 |  |
| St Mary's | 10/11/2021 15:00 | 10/11/2021 09:00 | 10/11/2021 15:00 | 0.0751 | 0.1145 | 19059335 | 1427 | 0 | 0 |  | 0 | 0 | 0 | 273.88 | 0 | 0 |  |
| Ffriddoedd site-Road | 10/11/2021 15:10 | 10/11/2021 09:10 | 10/11/2021 15:10 | 0.0700 | 0.0142 | 7733738 | 0 | 0 | 0 |  | 400.84 | 0 | 0 | 0 | 0 | 0 |  |
| Ffriddoedd site-Brailsford | 10/11/2021 15:20 | 10/11/2021 09:20 | 10/11/2021 15:20 | 0.0976 | 0.1380 | 10070691 | 176 | 0 | 0 |  | 0 | 0 | 0 | 0 | 0 | 0 |  |
| Ffriddoedd site-Reichel | 10/11/2021 15:30 | 10/11/2021 09:30 | 10/11/2021 15:30 | 0.0644 | 0.0276 | 4170866 | 0 | 0 | 0 |  | 0 | 0 | 0 | 0 | 0 | 0 |  |
| St Mary's | 11/11/2021 09:00 | 10/11/2021 15:00 | 11/11/2021 09:00 | 0.1398 |  | 4921897 | 161 | 2714.66 | 0 |  | 0 | 0 | 0 | 0 | 0 | 0 |  |
| Ffriddoedd site-Road | 11/11/2021 09:10 | 10/11/2021 15:10 | 11/11/2021 09:10 | 0.1830 |  | 3402605 | 0 | 0 | 0 |  | 0 | 0 | 0 | 0 | 0 | 0 |  |
| Ffriddoedd site-Brailsford | 11/11/2021 09:20 | 10/11/2021 15:10 | 11/11/2021 09:20 | 0.0884 | 0.1380 | 12043867 | 30 | 1826.88 | 0 |  | 0 | 0 | 0 | 0 | 0 | 0 |  |
| Ffriddoedd site-Reichel | 11/11/2021 09:30 | 10/11/2021 15:10 | 11/11/2021 09:30 | 0.0109 | 0.0503 | 930251 | 0 | 0 | 0 |  | 0 | 0 | 0 | 0 | 0 | 0 |  |
| St Mary's | 11/11/2021 15:00 | 11/11/2021 09:00 | 11/11/2021 15:00 | 0.0661 | 0.1458 | 5384886 | 242 | 2082.69 | 370.34 |  | 0 | 0 | 0 | 85.82 | 0 | 0 |  |
| Ffriddoedd site-Road | 11/11/2021 15:10 | 11/11/2021 09:10 | 11/11/2021 15:10 | 0.4503 |  | 1783812 | 32 | 0 | 0 |  | 0 | 0 | 0 | 0 | 0 | 0 |  |
| Ffriddoedd site-Brailsford | 11/11/2021 15:20 | 11/11/2021 09:20 | 11/11/2021 15:20 | 0.0927 | 0.1222 | 9315905 | 0 | 0 | 0 |  | 0 | 0 | 0 | 0 | 0 | 0 |  |
| Ffriddoedd site-Reichel | 11/11/2021 15:30 | 11/11/2021 09:30 | 11/11/2021 15:30 | 0.1112 |  | 8104044 | 40 | 0 | 0 |  | 0 | 0 | 0 | 0 | 0 | 0 |  |
| St Mary's | 12/11/2021 08:00 | 11/11/2021 15:00 | 12/11/2021 08:00 | 0.8142 |  | 62621580 | 4009 | 0 | 0 |  | 0 | 0 | 0 | 355.63 | 0 | 0 |  |
| Ffriddoedd site-Road | 12/11/2021 08:10 | 11/11/2021 15:10 | 12/11/2021 08:10 | 0.5397 |  | 58784862 | 0 | 0 | 0 |  | 7000.09 | 0 | 0 | 0 | 0 | 0 |  |
| Ffriddoedd site-Brailsford | 12/11/2021 08:20 | 11/11/2021 15:10 | 12/11/2021 08:20 | 0.3232 |  | 27126575 | 489 | 0 | 0 |  | 0 | 0 | 0 | 52.87 | 0 | 0 |  |
| Ffriddoedd site-Reichel | 12/11/2021 08:30 | 11/11/2021 15:10 | 12/11/2021 08:30 | 0.3023 |  | 39880796 | 0 | 0 | 0 |  | 0 | 0 | 0 | 0 | 0 | 0 |  |
| St Mary's | 12/11/2021 14:00 | 12/11/2021 08:00 | 12/11/2021 14:00 | 0.2226 |  | 121943150 | 6299 | 11383.99 | 0 |  | 0 | 0 | 0 | 554.47 | 0 | 0 |  |
| Ffriddoedd site-Road | 12/11/2021 14:10 | 12/11/2021 08:10 | 12/11/2021 14:10 | 0.0000 |  | 1618807 | 0 | 0 | 0 |  | 0 | 0 | 0 | 0 | 0 | 0 |  |
| Ffriddoedd site-Brailsford | 12/11/2021 14:20 | 12/11/2021 08:20 | 12/11/2021 14:20 | 0.1473 |  | 20168524 | 0 | 290.44 | 391.78 |  | 0 | 0 | 0 | 0 | 0 | 0 |  |
| Ffriddoedd site-Reichel | 12/11/2021 14:30 | 12/11/2021 08:30 | 12/11/2021 14:30 | 0.2503 |  | 28101188 | 0 | 0 | 0 |  | 0 | 0 | 0 | 0 | 0 | 0 |  |
| St Mary's | 08/11/2021 15:00 | 08/11/2021 09:00 | 08/11/2021 15:00 | 0.0072 | 0.0852 | 4558606 | 0 | 0 | 0 |  | 0 | 0 | 0 | 0 | 0 | 0 |  |
| Ffriddoedd site-Road | 08/11/2021 15:10 | 08/11/2021 09:10 | 08/11/2021 15:10 | 0.0559 |  | 21794283 | 96 | 0 | 0 |  | 0 | 0 | 0 | 0 | 0 | 0 |  |
| Ffriddoedd site-Brailsford | 08/11/2021 15:20 | 08/11/2021 09:20 | 08/11/2021 15:20 | 0.1873 |  | 64528490 | 64 | 1749.53 | 267.68 |  | 0 | 0 | 0 | 0 | 0 | 0 |  |
| Ffriddoedd site-Reichel | 08/11/2021 15:30 | 08/11/2021 09:30 | 08/11/2021 15:30 | 0.0063 |  | 10249600 | 0 | 0 | 0 |  | 0 | 0 | 0 | 0 | 0 | 0 |  |
| St Mary's | 15/11/2021 15:00 | 15/11/2021 09:00 | 15/11/2021 15:00 | 0.2281 |  | 62685628 | 897 | 1724.68 | 64.85 |  | 0 | 0 | 0 | 246.8 | 0 | 61.9 |  |
| Ffriddoedd site-Road | 15/11/2021 15:10 | 15/11/2021 09:10 | 15/11/2021 15:10 | 0.1308 |  | 14229701 | 1154 | 0 | 0 |  | 103.34 | 0 | 0 | 644.65 | 0 | 0 |  |
| Ffriddoedd site-Brailsford | 15/11/2021 15:20 | 15/11/2021 09:20 | 15/11/2021 15:20 | 0.0899 | 0.1139 | 48452486 | 0 | 0 | 130.62 |  | 0 | 0 | 0 | 0 | 0 | 0 |  |
| Ffriddoedd site-Reichel | 15/11/2021 15:30 | 15/11/2021 09:30 | 15/11/2021 15:30 | 0.1708 |  | 22237945 | 0 | 0 | 0 |  | 0 | 0 | 0 | 0 | 0 | 0 |  |
| St Mary's | 17/11/2021 15:00 | 17/11/2021 09:00 | 17/11/2021 15:00 | 0.5459 |  | 20927696 | 120608 | 0 | 352.26 |  | 0 | 0 | 2613.56 | 17429.17 | 21462.36 | 0 |  |
| Ffriddoedd site-Road | 17/11/2021 15:10 | 17/11/2021 09:10 | 17/11/2021 15:10 | 0.1856 |  | 17854671 | 67 | 0 | 0 |  | 0 | 0 | 0 | 0 | 0 | 0 |  |
| Ffriddoedd site-Brailsford | 17/11/2021 15:20 | 17/11/2021 09:20 | 17/11/2021 15:20 | 0.2905 |  | 49312125 | 0 | 0 | 0 |  | 0 | 0 | 0 | 0 | 0 | 0 |  |
| Ffriddoedd site-Reichel | 17/11/2021 15:30 | 17/11/2021 09:30 | 17/11/2021 15:30 | 0.3115 |  | 46970047 | 37 | 0 | 0 |  | 7726.48 | 0 | 0 | 0 | 0 | 0 |  |
| St Mary's | 18/11/2021 09:00 | 17/11/2021 15:00 | 18/11/2021 09:00 | 0.3850 |  | 0 | 49418 | 296.71 | 0 |  | 233.7 | 0 | 1777.92 | 8041.44 | 10002.4 | 1787.54 |  |
| Ffriddoedd site-Road | 18/11/2021 09:10 | 17/11/2021 15:10 | 18/11/2021 09:10 | 0.6222 |  | 51271542 | 450 | 0 | 0 |  | 570.59 | 0 | 0 | 118.25 | 0 | 0 |  |
| Ffriddoedd site-Brailsford | 18/11/2021 09:20 | 17/11/2021 15:20 | 18/11/2021 09:20 | 0.4303 |  | 37000356 | 901 | 0 | 167.51 |  | 0 | 0 | 0 | 0 | 0 | 0 |  |
| Ffriddoedd site-Reichel | 18/11/2021 09:30 | 17/11/2021 15:30 | 18/11/2021 09:30 | 0.3375 |  | 22343888 | 35 | 0 | 0 |  | 1253.36 | 0 | 0 | 0 | 0 | 0 |  |
| St Mary's | 18/11/2021 15:00 | 18/11/2021 09:00 | 18/11/2021 15:00 | 0.2226 |  | 12044064 | 32593 | 0 | 0 |  | 0 | 0 | 0 | 3395.9 | 6992.13 | 0 |  |
| Ffriddoedd site-Road | 18/11/2021 15:10 | 18/11/2021 09:10 | 18/11/2021 15:10 | 0.8521 |  | 28853963 | 0 | 0 | 0 |  | 0 | 0 | 0 | 0 | 0 | 0 |  |
| Ffriddoedd site-Brailsford | 18/11/2021 15:20 | 18/11/2021 09:20 | 18/11/2021 15:20 | 0.0731 |  | 5390672 | 0 | 0 | 22.9 |  | 188.86 | 0 | 0 | 0 | 0 | 0 |  |
| Ffriddoedd site-Reichel | 18/11/2021 15:30 | 18/11/2021 09:30 | 18/11/2021 15:30 | 0.3715 |  | 9004039 | 0 | 0 | 318.93 |  | 0 | 0 | 0 | 0 | 0 | 0 |  |
| St Mary's | 19/11/2021 08:00 | 18/11/2021 15:00 | 19/11/2021 08:00 | 0.6100 |  | 12852605 | 41607 | 948.59 | 380.11 |  | 338.23 | 0 | 0 | 3935.07 | 7281.63 | 0 |  |
| Ffriddoedd site-Road | 19/11/2021 08:10 | 18/11/2021 15:10 | 19/11/2021 08:10 | 0.2500 |  | 12086640 | 8264 | 0 | 0 |  | 0 | 0 | 0 | 1020.55 | 0 | 0 |  |
| Ffriddoedd site-Brailsford | 19/11/2021 08:20 | 18/11/2021 15:20 | 19/11/2021 08:20 | 0.2475 |  | 28946350 | 0 | 0 | 182.62 |  | 0 | 0 | 0 | 0 | 0 | 0 |  |
| Ffriddoedd site-Reichel | 19/11/2021 08:30 | 18/11/2021 15:30 | 19/11/2021 08:30 | 0.1526 |  | 6467115 | 0 | 0 | 0 |  | 8606.86 | 0 | 0 | 0 | 0 | 0 |  |
| St Mary's | 19/11/2021 14:00 | 19/11/2021 08:00 | 19/11/2021 14:00 | 0.3035 |  | 15218157 | 53 | 0 | 0 |  | 0 | 0 | 0 | 0 | 0 | 0 |  |
| Ffriddoedd site-Road | 19/11/2021 14:10 | 19/11/2021 08:10 | 19/11/2021 14:10 | 1.5828 |  | 5171215 | 0 | 0 | 0 |  | 0 | 0 | 0 | 0 | 0 | 0 |  |
| Ffriddoedd site-Brailsford | 19/11/2021 14:20 | 19/11/2021 08:20 | 19/11/2021 14:20 | 0.0216 |  | 37731933 | 0 | 0 | 0 |  | 512.3 | 0 | 0 | 0 | 0 | 0 |  |
| Ffriddoedd site-Reichel | 19/11/2021 14:30 | 19/11/2021 08:30 | 19/11/2021 14:30 | 0.3490 |  | 8232444 | 0 | 0 | 0 |  | 2275.7 | 0 | 0 | 0 | 0 | 0 |  |
| St Mary's | 22/11/2021 15:00 | 22/11/2021 09:00 | 22/11/2021 15:00 | 0.1245 |  | 386988035 | 146 | 249.8 | 497.39 |  | 28299.6 | 0 | 0 | 0 | 0 | 0 |  |
| Ffriddoedd site-Road | 22/11/2021 15:10 | 22/11/2021 09:00 | 22/11/2021 15:10 | 0.1085 |  | 130710050 | 38 | 0 | 0 |  | 0 | 0 | 0 | 0 | 0 | 0 |  |
| Ffriddoedd site-Brailsford | 22/11/2021 15:20 | 22/11/2021 09:00 | 22/11/2021 15:20 | 0.0159 |  | 150971275 | 150 | 0 | 0 |  | 297.75 | 0 | 0 | 68.16 | 0 | 0 |  |
| Ffriddoedd site-Reichel | 22/11/2021 15:30 | 22/11/2021 09:00 | 22/11/2021 15:30 | 0.2330 |  | 46970788 | 4302 | 0 | 134.8 |  | 351325.2 | 0 | 0 | 428.28 | 0 | 0 |  |
| St Mary's | 23/11/2021 09:00 | 22/11/2021 15:00 | 23/11/2021 09:00 | 0.2044 |  | 193722000 | 127246 | 0 | 0 |  | 1071.55 | 0 | 5951.87 | 20505.73 | 18826.86 | 0 |  |
| Ffriddoedd site-Road | 23/11/2021 09:10 | 22/11/2021 15:10 | 23/11/2021 09:10 | 0.2200 |  | 130510080 | 0 | 0 | 0 |  | 0 | 0 | 0 | 0 | 0 | 0 |  |
| Ffriddoedd site-Brailsford | 23/11/2021 09:20 | 22/11/2021 15:20 | 23/11/2021 09:20 | 0.1529 |  | 249622290 | 1159 | 0 | 0 |  | 8547.06 | 0 | 0 | 277.09 | 0 | 0 |  |
| Ffriddoedd site-Reichel | 23/11/2021 09:30 | 22/11/2021 15:30 | 23/11/2021 09:30 | 0.1386 |  | 175021635 | 44 | 0 | 0 |  | 31524.77 | 0 | 0 | 0 | 0 | 0 |  |
| St Mary's | 23/11/2021 15:00 | 23/11/2021 09:00 | 23/11/2021 15:00 | 0.1214 |  | 92716902 | 17694 | 0 | 0 |  | 7280.75 | 0 | 0 | 2829.52 | 5239.04 | 0 |  |
| Ffriddoedd site-Road | 23/11/2021 15:10 | 23/11/2021 09:10 | 23/11/2021 15:10 | 0.1791 |  | 73867450 | 39974 | 0 | 0 |  | 0 | 0 | 0 | 2945.67 | 5951.33 | 0 |  |
| Ffriddoedd site-Brailsford | 23/11/2021 15:20 | 23/11/2021 09:20 | 23/11/2021 15:20 | 0.0529 |  | 221753945 | 550 | 0 | 0 |  | 2696.18 | 0 | 0 | 85.44 | 0 | 0 |  |
| Ffriddoedd site-Reichel | 23/11/2021 15:30 | 23/11/2021 09:30 | 23/11/2021 15:30 | 0.2069 |  | 129432890 | 43 | 0 | 0 |  | 26746.19 | 0 | 0 | 0 | 0 | 0 |  |
| St Mary's | 24/11/2021 09:00 | 23/11/2021 15:00 | 24/11/2021 09:00 | 0.1539 |  | 170960880 | 278 | 0 | 0 |  | 15780.14 | 0 | 0 | 29.39 | 0 | 0 |  |
| Ffriddoedd site-Road | 24/11/2021 09:10 | 23/11/2021 15:10 | 24/11/2021 09:10 | 0.1251 |  | 208612505 | 1122 | 0 | 0 |  | 202.72 | 0 | 0 | 51.03 | 0 | 0 |  |
| Ffriddoedd site-Brailsford | 24/11/2021 09:20 | 23/11/2021 15:20 | 24/11/2021 09:20 | 0.1520 |  | 474855850 | 108 | 149.47 | 160.18 |  | 7824.62 | 0 | 0 | 62.37 | 0 | 0 |  |
| Ffriddoedd site-Reichel | 24/11/2021 09:30 | 23/11/2021 15:30 | 24/11/2021 09:30 | 0.0665 |  | 115128430 | 2080 | 0 | 0 |  | 16088.03 | 0 | 0 | 356.15 | 0 | 0 |  |
| St Mary's | 24/11/2021 15:00 | 24/11/2021 09:00 | 24/11/2021 15:00 | 0.4238 |  | 74274636 | 171 | 0 | 0 |  | 2676.67 | 0 | 0 | 0 | 0 | 0 |  |
| Ffriddoedd site-Road | 24/11/2021 15:10 | 24/11/2021 09:10 | 24/11/2021 15:10 | 0.4852 |  | 4036116 | 0 | 0 | 35.29 |  | 105.41 | 0 | 0 | 0 | 0 | 0 |  |
| Ffriddoedd site-Brailsford | 24/11/2021 15:20 | 24/11/2021 09:20 | 24/11/2021 15:20 | 0.2740 |  | 101900015 | 0 | 0 | 0 |  | 4273.05 | 0 | 0 | 0 | 0 | 0 |  |
| Ffriddoedd site-Reichel | 24/11/2021 15:30 | 24/11/2021 09:30 | 24/11/2021 15:30 | 0.9566 |  | 66666542 | 0 | 0 | 0 |  | 23042.27 | 0 | 0 | 0 | 0 | 0 |  |
| St Mary's | 25/11/2021 09:00 | 24/11/2021 15:00 | 25/11/2021 09:00 | 0.4972 |  | 101469895 | 0 | 0 | 0 |  | 5935.57 | 0 | 0 | 0 | 0 | 0 |  |
| Ffriddoedd site-Road | 25/11/2021 09:10 | 24/11/2021 15:10 | 25/11/2021 09:10 | 0.2910 |  | 33390950 | 0 | 0 | 0 |  | 20.99 | 0 | 0 | 28.34 | 0 | 0 |  |
| Ffriddoedd site-Brailsford | 25/11/2021 09:20 | 24/11/2021 15:20 | 25/11/2021 09:20 | 0.3112 |  | 186080815 | 500 | 0 | 0 |  | 3153.96 | 0 | 0 | 96.91 | 0 | 0 |  |
| Ffriddoedd site-Reichel | 25/11/2021 09:30 | 24/11/2021 15:30 | 25/11/2021 09:30 | 0.7538 |  | 142105445 | 2586 | 0 | 0 |  | 8841.66 | 0 | 0 | 358.62 | 607.49 | 0 |  |
| St Mary's | 25/11/2021 15:00 | 25/11/2021 09:00 | 25/11/2021 15:00 | 31.5936 |  | 604490125 | 15615 | 0 | 0 |  | 4231.3 | 0 | 944.09 | 3042.7 | 3690.02 | 0 |  |
| Ffriddoedd site-Road | 25/11/2021 15:10 | 25/11/2021 09:10 | 25/11/2021 15:10 | 0.0000 | 13.8010 | 421226050 | 0 | 0 | 0 |  | 37.67 | 0 | 0 | 0 | 0 | 0 |  |
| Ffriddoedd site-Brailsford | 25/11/2021 15:20 | 25/11/2021 09:20 | 25/11/2021 15:20 | 0.9156 |  | 260944200 | 32 | 0 | 0 |  | 1677.49 | 0 | 0 | 0 | 0 | 0 |  |
| Ffriddoedd site-Reichel | 25/11/2021 15:30 | 25/11/2021 09:30 | 25/11/2021 15:30 | 0.0000 | 0.0000 | 38406817 | 257 | 0 | 0 |  | 4922.73 | 0 | 0 | 20.55 | 0 | 0 |  |
| St Mary's | 26/11/2021 08:00 | 25/11/2021 15:00 | 26/11/2021 08:00 | 0.0000 | 0.0000 | 225505355 | 34 | 0 | 5.55 |  | 6227.3 | 0 | 0 | 0 | 0 | 0 |  |
| Ffriddoedd site-Road | 26/11/2021 08:10 | 25/11/2021 15:10 | 26/11/2021 08:10 | 0.0000 | 0.0000 | 37777808 | 446 | 0 | 40.37 |  | 0 | 0 | 0 | 0 | 0 | 0 |  |
| Ffriddoedd site-Brailsford | 26/11/2021 08:20 | 25/11/2021 15:20 | 26/11/2021 08:20 | 0.7392 |  | 173217285 | 106 | 0 | 0 |  | 4293.28 | 0 | 0 | 50.64 | 0 | 0 |  |
| Ffriddoedd site-Reichel | 26/11/2021 08:30 | 25/11/2021 15:30 | 26/11/2021 08:30 | 0.0000 | 0.0000 | 77339918 | 0 | 0 | 0 |  | 7900.98 | 0 | 0 | 0 | 0 | 0 |  |
| St Mary's | 26/11/2021 14:00 | 26/11/2021 08:00 | 26/11/2021 14:00 | 0.0000 | 14.2072 | 29985085 | 0 | 0 | 0 |  | 183.04 | 0 | 0 | 0 | 0 | 0 |  |
| Ffriddoedd site-Road | 26/11/2021 14:10 | 26/11/2021 08:10 | 26/11/2021 14:10 | 9.4604 |  | 48367181 | 393 | 0 | 0 |  | 0 | 0 | 0 | 40.77 | 0 | 0 |  |
| Ffriddoedd site-Brailsford | 26/11/2021 14:20 | 26/11/2021 08:20 | 26/11/2021 14:20 | 0.0000 | 0.0000 | 54444720 | 0 | 0 | 0 |  | 1721.33 | 0 | 0 | 0 | 0 | 0 |  |
| Ffriddoedd site-Reichel | 26/11/2021 14:30 | 26/11/2021 08:30 | 26/11/2021 14:30 | 2.4327 |  | 143878090 | 0 | 0 | 0 |  | 1359.71 | 0 | 0 | 0 | 0 | 0 |  |
| St Mary's | 29/11/2021 15:00 | 29/11/2021 09:00 | 29/11/2021 15:00 | 0.3742 |  | 1299792600 | 1482 | 0 | 0 |  | 297.08 | 0 | 0 | 70.38 | 0 | 0 |  |
| Ffriddoedd site-Road | 29/11/2021 15:10 | 29/11/2021 09:00 | 29/11/2021 15:10 | 0.6035 |  | 46472165 | 83 | 0 | 0 |  | 0 | 0 | 0 | 32.24 | 0 | 0 |  |
| Ffriddoedd site-Brailsford | 29/11/2021 15:20 | 29/11/2021 09:00 | 29/11/2021 15:20 | 0.4914 |  | 240310120 | 3081 | 1233.26 | 0 |  | 17562.58 | 0 | 0 | 168.87 | 0 | 0 |  |
| Ffriddoedd site-Reichel | 29/11/2021 15:30 | 29/11/2021 09:00 | 29/11/2021 15:30 | 0.3659 |  | 402054325 | 584 | 0 | 0 |  | 5239.64 | 0 | 0 | 36.42 | 0 | 0 |  |
| St Mary's | 30/11/2021 09:00 | 29/11/2021 15:00 | 30/11/2021 09:00 | 0.1747 |  | 354943575 | 0 | 0 | 0 |  | 3691.69 | 0 | 0 | 0 | 0 | 0 |  |
| Ffriddoedd site-Road | 30/11/2021 09:10 | 29/11/2021 15:10 | 30/11/2021 09:10 | 0.2802 |  | 194520400 | 1714 | 0 | 0 |  | 58.35 | 0 | 0 | 113.22 | 0 | 0 |  |
| Ffriddoedd site-Brailsford | 30/11/2021 09:20 | 29/11/2021 15:20 | 30/11/2021 09:20 | 0.2889 |  | 807689100 | 79 | 17539.27 | 0 |  | 7737.87 | 0 | 0 | 48.87 | 0 | 0 |  |
| Ffriddoedd site-Reichel | 30/11/2021 09:30 | 29/11/2021 15:30 | 30/11/2021 09:30 | 0.1695 |  | 807252175 | 65 | 0 | 0 |  | 679.76 | 0 | 0 | 0 | 0 | 0 |  |
| St Mary's | 30/11/2021 15:00 | 30/11/2021 09:00 | 30/11/2021 15:00 |  |  | 2123740800 | 0 | 0 | 0 |  | 150.5 | 0 | 0 | 49.6 | 0 | 0 |  |
| Ffriddoedd site-Road | 30/11/2021 15:10 | 30/11/2021 09:10 | 30/11/2021 15:10 |  |  | 1925338600 | 0 | 0 | 0 |  | 156.99 | 0 | 0 | 12.96 | 0 | 0 |  |
| Ffriddoedd site-Brailsford | 30/11/2021 15:20 | 30/11/2021 09:20 | 30/11/2021 15:20 |  |  | 1532501750 | 0 | 2754.07 | 0 |  | 2303.5 | 0 | 0 | 0 | 0 | 0 |  |
| Ffriddoedd site-Reichel | 30/11/2021 15:30 | 30/11/2021 09:30 | 30/11/2021 15:30 |  |  | 4071200600 | 0 | 0 | 0 |  | 7425.68 | 0 | 0 | 65.46 | 0 | 0 |  |
| St Mary's | 01/12/2021 09:00 | 30/11/2021 15:00 | 01/12/2021 09:00 |  |  | 694665875 | 0 | 0 | 0 |  | 6437.96 | 0 | 0 | 0 | 0 | 0 |  |
| Ffriddoedd site-Road | 01/12/2021 09:10 | 30/11/2021 15:10 | 01/12/2021 09:10 |  |  | 37890655 | 0 | 0 | 0 |  | 11557.34 | 0 | 0 | 0 | 0 | 0 |  |
| Ffriddoedd site-Brailsford | 01/12/2021 09:20 | 30/11/2021 15:20 | 01/12/2021 09:20 |  |  | 7581424400 | 0 | 194.46 | 0 |  | 8149.71 | 0 | 0 | 0 | 0 | 0 |  |
| Ffriddoedd site-Reichel | 01/12/2021 09:30 | 30/11/2021 15:30 | 01/12/2021 09:30 |  |  | 262907960 | 0 | 0 | 0 |  | 1763.56 | 0 | 0 | 0 | 0 | 0 |  |
| St Mary's | 01/12/2021 15:00 | 01/12/2021 09:00 | 01/12/2021 15:00 | 0.1089 |  | 244278255 | 79 | 0 | 0 |  | 0 | 0 | 0 | 0 | 0 | 0 |  |
| Ffriddoedd site-Road | 01/12/2021 15:10 | 01/12/2021 09:10 | 01/12/2021 15:10 | 0.7046 |  | 185074795 | 90 | 0 | 5.81 |  | 5422.24 | 0 | 0 | 9.8 | 0 | 0 |  |
| Ffriddoedd site-Brailsford | 01/12/2021 15:20 | 01/12/2021 09:20 | 01/12/2021 15:20 | 0.3195 |  | 221668525 | 25 | 0 | 0 |  | 1111.03 | 0 | 0 | 0 | 0 | 0 |  |
| Ffriddoedd site-Reichel | 01/12/2021 15:30 | 01/12/2021 09:30 | 01/12/2021 15:30 | 0.2558 |  | 294763370 | 0 | 0 | 0 |  | 3137.06 | 0 | 0 | 0 | 0 | 0 |  |
| St Mary's | 02/12/2021 09:00 | 01/12/2021 15:00 | 02/12/2021 09:00 | 0.4829 |  | 1586290950 | 402 | 0 | 0 |  | 1361.09 | 0 | 0 | 124.46 | 0 | 0 |  |
| Ffriddoedd site-Road | 02/12/2021 09:10 | 01/12/2021 15:10 | 02/12/2021 09:10 | 2.2567 |  | 198228090 | 89 | 0 | 0 |  | 450.65 | 0 | 0 | 0 | 0 | 0 |  |
| Ffriddoedd site-Brailsford | 02/12/2021 09:20 | 01/12/2021 15:20 | 02/12/2021 09:20 | 1.0579 |  | 1831110200 | 0 | 0 | 0 |  | 676.15 | 0 | 0 | 0 | 0 | 0 |  |
| St Mary's | 02/12/2021 15:00 | 02/12/2021 09:00 | 02/12/2021 15:00 | 0.0628 | 0.0652 | 802804350 | 1883 | 0 | 0 |  | 1003.19 | 0 | 0 | 311.34 | 0 | 0 |  |
| Ffriddoedd site-Road | 02/12/2021 15:10 | 02/12/2021 09:10 | 02/12/2021 15:10 | 0.3862 |  | 308694670 | 0 | 0 | 0 |  | 2128.16 | 0 | 0 | 0 | 0 | 0 |  |
| Ffriddoedd site-Brailsford | 02/12/2021 15:20 | 02/12/2021 09:20 | 02/12/2021 15:20 | 0.2475 |  | 996899500 | 0 | 0 | 18.93 |  | 1305.69 | 0 | 0 | 34.31 | 0 | 0 |  |
| Ffriddoedd site-Reichel | 02/12/2021 15:20 | 02/12/2021 09:30 | 02/12/2021 15:30 | 0.1569 | 0.0166 | 1779163200 | 54 | 0 | 0 |  | 2448.88 | 0 | 0 | 14.84 | 0 | 0 |  |
| St Mary's | 03/12/2021 08:00 | 02/12/2021 15:00 | 03/12/2021 08:00 | 0.0432 | 0.0220 | 1069230200 | 163 | 0 | 0 |  | 1521.67 | 0 | 0 | 62.17 | 0 | 0 |  |
| Ffriddoedd site-Road | 03/12/2021 08:10 | 02/12/2021 15:10 | 03/12/2021 08:10 | 0.6098 |  | 122722105 | 29 | 0 | 0 |  | 126.89 | 0 | 0 | 28.05 | 0 | 0 |  |
| Ffriddoedd site-Brailsford | 03/12/2021 08:20 | 02/12/2021 15:20 | 03/12/2021 08:20 | 0.2147 |  | 812983975 | 17 | 0 | 0 |  | 672.91 | 0 | 0 | 0 | 0 | 0 |  |
| Ffriddoedd site-Reichel | 03/12/2021 08:30 | 02/12/2021 15:30 | 03/12/2021 08:30 | 0.4436 |  | 384575135 | 880 | 0 | 0 |  | 1033.94 | 0 | 0 | 123.38 | 0 | 0 |  |
| St Mary's | 03/12/2021 14:00 | 03/12/2021 08:00 | 03/12/2021 14:00 | 0.1955 | 0.2787 | 752440875 | 8118 | 0 | 0 |  | 29.21 | 0 | 0 | 655.8 | 0 | 0 |  |
| Ffriddoedd site-Road | 03/12/2021 14:10 | 03/12/2021 08:10 | 03/12/2021 14:10 | 0.2718 |  | 217772065 | 85 | 0 | 54.22 |  | 194.17 | 0 | 0 | 0 | 0 | 0 |  |
| Ffriddoedd site-Brailsford | 03/12/2021 14:20 | 03/12/2021 08:20 | 03/12/2021 14:20 | 0.0862 | 0.0000 | 1889254200 | 0 | 0 | 0 |  | 441.99 | 0 | 0 | 0 | 0 | 0 |  |
| Ffriddoedd site-Reichel | 03/12/2021 14:30 | 03/12/2021 08:30 | 03/12/2021 14:30 | 0.2078 |  | 400288385 | 1208 | 0 | 0 |  | 280.42 | 0 | 0 | 71.05 | 0 | 0 |  |
| Ffriddoedd site-Reichel | 02/12/2021 09:30 | 01/12/2021 15:30 | 02/12/2021 09:30 | 0.6331 |  | 100873730 | 0 | 0 | 0 |  | 1063.9 | 0 | 0 | 0 | 0 | 0 |  |
| St Mary's | 06/12/2021 15:00 | 06/12/2021 09:00 | 06/12/2021 15:00 | 3.4075 |  | 1791654 | 4597 | 0 | 0 |  | 0 | 0 | 0 | 502.81 | 0 | 0 |  |
| Ffriddoedd site-Road | 06/12/2021 15:10 | 06/12/2021 09:00 | 06/12/2021 15:10 | 6.1385 |  | 748780 | 1203 | 0 | 22.31 |  | 1858.76 | 0 | 0 | 132.81 | 0 | 0 |  |
| Ffriddoedd site-Brailsford | 06/12/2021 15:20 | 06/12/2021 09:00 | 06/12/2021 15:20 | 0.9063 | 0.0270 | 3060291 | 1007 | 774.66 | 0 |  | 0 | 0 | 0 | 315.07 | 0 | 0 |  |
| Ffriddoedd site-Reichel | 06/12/2021 15:30 | 06/12/2021 09:00 | 06/12/2021 15:30 | 1.3604 |  | 1087601 | 436 | 0 | 15.34 |  | 160.42 | 0 | 0 | 124.78 | 0 | 0 |  |
| St Mary's | 07/12/2021 09:00 | 06/12/2021 15:00 | 07/12/2021 09:00 | 1.9508 |  | 3403684 | 0 | 0 | 0 |  | 0 | 0 | 0 | 0 | 0 | 0 |  |
| Ffriddoedd site-Road | 07/12/2021 09:10 | 06/12/2021 15:10 | 07/12/2021 09:10 | 0.7948 | 0.0372 | 990699 | 4112 | 0 | 0 |  | 1803.31 | 0 | 0 | 690.75 | 0 | 0 |  |
| Ffriddoedd site-Brailsford | 07/12/2021 09:20 | 06/12/2021 15:20 | 07/12/2021 09:20 | 1.2591 |  | 3527388 | 374 | 0 | 0 |  | 401.63 | 0 | 0 | 101.94 | 0 | 0 |  |
| Ffriddoedd site-Reichel | 07/12/2021 09:30 | 06/12/2021 15:30 | 07/12/2021 09:30 | 3.9074 |  | 2647415 | 165 | 0 | 13.07 |  | 0 | 0 | 0 | 87.29 | 0 | 0 |  |
| St Mary's | 07/12/2021 15:00 | 07/12/2021 09:00 | 07/12/2021 15:00 | 0.5172 | 0.0129 | 2509135 | 0 | 0 | 0 |  | 0 | 0 | 0 | 0 | 0 | 0 |  |
| Ffriddoedd site-Road | 07/12/2021 15:10 | 07/12/2021 09:10 | 07/12/2021 15:10 | 14.2150 |  | 1304546 | 355413 | 0 | 0 |  | 4379.77 | 0 | 13501.52 | 41129.96 | 29917.88 | 0 |  |
| Ffriddoedd site-Brailsford | 07/12/2021 15:20 | 07/12/2021 09:20 | 07/12/2021 15:20 | 1.3385 |  | 7561895 | 1477 | 495.25 | 111.76 |  | 0 | 0 | 0 | 179.17 | 0 | 0 |  |
| Ffriddoedd site-Reichel | 07/12/2021 15:30 | 07/12/2021 09:30 | 07/12/2021 15:30 | 0.0000 | 0.0000 | 1384855 | 0 | 0 | 0 |  | 0 | 0 | 0 | 0 | 0 | 0 |  |
| St Mary's | 08/12/2021 09:00 | 07/12/2021 15:00 | 08/12/2021 09:00 | 1.7619 |  | 1688196 | 3419 | 0 | 6.29 |  | 19.08 | 0 | 0 | 521.07 | 0 | 0 |  |
| Ffriddoedd site-Road | 08/12/2021 09:10 | 07/12/2021 15:10 | 08/12/2021 09:10 | 7.5098 |  | 1991258 | 2944 | 0 | 0 |  | 2779.23 | 0 | 0 | 409.84 | 0 | 0 |  |
| Ffriddoedd site-Brailsford | 08/12/2021 09:20 | 07/12/2021 15:20 | 08/12/2021 09:20 | 2.5766 |  | 5658216 | 502 | 0 | 3.08 |  | 0 | 0 | 0 | 113.9 | 0 | 0 |  |
| Ffriddoedd site-Reichel | 08/12/2021 09:30 | 07/12/2021 15:30 | 08/12/2021 09:30 | 1.4137 | 0.0540 | 1249491 | 0 | 0 | 0 |  | 0 | 0 | 0 | 0 | 0 | 0 |  |
| St Mary's | 08/12/2021 15:00 | 08/12/2021 09:00 | 08/12/2021 15:00 | 1.5307 |  | 1904153 | 726 | 0 | 0 |  | 0 | 0 | 0 | 73.78 | 0 | 0 |  |
| Ffriddoedd site-Road | 08/12/2021 15:10 | 08/12/2021 09:10 | 08/12/2021 15:10 | 8.4130 |  | 214830 | 17831 | 0 | 0 |  | 719.99 | 0 | 529.84 | 2494.65 | 3377.88 | 0 |  |
| Ffriddoedd site-Brailsford | 08/12/2021 15:20 | 08/12/2021 09:20 | 08/12/2021 15:20 | 1.4763 |  | 4555518 | 288 | 0 | 2.11 |  | 0 | 0 | 0 | 0 | 0 | 0 |  |
| Ffriddoedd site-Reichel | 08/12/2021 15:30 | 08/12/2021 09:30 | 08/12/2021 15:30 | 3.7017 |  | 1699280 | 26 | 0 | 31.59 |  | 0 | 0 | 0 | 0 | 0 | 0 |  |
| St Mary's | 13/12/2021 15:00 | 13/12/2021 09:00 | 13/12/2021 15:00 | 0.7984 |  | 913053 | 339 | 0 | 0 | 1742.16 | 0 | 0 | 0 | 40.04 | 0 | 0 |  |
| Ffriddoedd site-Road | 13/12/2021 15:10 | 13/12/2021 09:00 | 13/12/2021 15:10 | 0.7346 |  | 155972 | 1434 | 0 | 0 | 0 | 404.35 | 0 | 0 | 145.35 | 0 | 0 |  |
| Ffriddoedd site-Brailsford | 13/12/2021 15:20 | 13/12/2021 09:00 | 13/12/2021 15:20 | 1.3217 |  | 1584028 | 13 | 0 | 0 | 0 | 0 | 0 | 0 | 0 | 0 | 0 |  |
| Ffriddoedd site-Reichel | 13/12/2021 15:30 | 13/12/2021 09:00 | 13/12/2021 15:30 | 0.9948 |  | 4484873 | 0 | 0 | 0 | 0 | 0 | 0 | 0 | 0 | 0 | 0 |  |
| St Mary's | 14/12/2021 09:00 | 13/12/2021 15:00 | 14/12/2021 09:00 | 0.4465 |  | 1207521 | 186 | 0 | 0 | 0 | 0 | 0 | 0 | 118.69 | 0 | 0 |  |
| Ffriddoedd site-Road | 14/12/2021 09:10 | 13/12/2021 15:10 | 14/12/2021 09:10 | 0.3832 |  | 159146 | 1093 | 0 | 0 | 0 | 3748.72 | 0 | 0 | 201.45 | 0 | 0 |  |
| Ffriddoedd site-Brailsford | 14/12/2021 09:20 | 13/12/2021 15:20 | 14/12/2021 09:20 | 0.2614 |  | 2664049 | 0 | 0 | 0 | 0 | 0 | 0 | 0 | 39.45 | 0 | 0 |  |
| Ffriddoedd site-Reichel | 14/12/2021 09:30 | 13/12/2021 15:30 | 14/12/2021 09:30 | 0.1570 |  | 1730402 | 0 | 0 | 0 | 0 | 0 | 0 | 0 | 0 | 0 | 0 |  |
| St Mary's | 14/12/2021 15:00 | 14/12/2021 09:00 | 14/12/2021 15:00 | 0.0000 |  | 939234 | 364 | 0 | 0 | 0 | 0 | 0 | 0 | 0 | 0 | 0 |  |
| Ffriddoedd site-Road | 14/12/2021 15:10 | 14/12/2021 09:10 | 14/12/2021 15:10 | 0.4935 |  | 516340 | 8031 | 0 | 0 | 0 | 0 | 0 | 0 | 1078.54 | 0 | 0 |  |
| Ffriddoedd site-Brailsford | 14/12/2021 15:20 | 14/12/2021 09:20 | 14/12/2021 15:20 | 0.2515 |  | 6161839 | 914 | 0 | 0 | 0 | 0 | 0 | 0 | 121.42 | 0 | 0 |  |
| Ffriddoedd site-Reichel | 14/12/2021 15:30 | 14/12/2021 09:30 | 14/12/2021 15:30 | 0.5061 |  | 2336556 | 0 | 0 | 0 | 0 | 0 | 0 | 0 | 0 | 0 | 0 |  |
| St Mary's | 15/12/2021 09:00 | 14/12/2021 15:00 | 15/12/2021 09:00 | 0.4764 |  | 1211906 | 3974 | 0 | 0 | 0 | 0 | 0 | 0 | 499.01 | 0 | 0 |  |
| Ffriddoedd site-Road | 15/12/2021 09:10 | 14/12/2021 15:10 | 15/12/2021 09:10 | 0.3593 |  | 1524694 | 195 | 0 | 0 | 0 | 0 | 0 | 0 | 43.2 | 0 | 0 |  |
| Ffriddoedd site-Brailsford | 15/12/2021 09:20 | 14/12/2021 15:20 | 15/12/2021 09:20 | 0.0543 |  | 1396194 | 0 | 0 | 0 | 0 | 0 | 0 | 0 | 0 | 0 | 0 |  |
| Ffriddoedd site-Reichel | 15/12/2021 09:30 | 14/12/2021 15:30 | 15/12/2021 09:30 | 0.0375 |  | 1872423 | 0 | 0 | 0 | 0 | 0 | 0 | 0 | 0 | 0 | 0 |  |
| St Mary's | 10/01/2022 15:00 | 10/01/2022 09:00 | 10/01/2022 15:00 |  | 0.2958 | 3592692 | 3398 | 0 | 0 | 0 | 0 | 0 |  | 0 |  |  | 752.55 |
| Ffriddoedd site-Road | 10/01/2022 15:00 | 10/01/2022 09:10 | 10/01/2022 15:00 |  |  | 692725 | 0 | 0 | 0 | 0 | 0 | 0 |  | 0 |  |  | 0 |
| Ffriddoedd site-Brailsford | 10/01/2022 15:00 | 10/01/2022 09:20 | 10/01/2022 15:00 |  | 0.0406 | 1674024 | 438 | 0 | 0 | 0 | 0 | 0 |  | 0 |  |  | 0 |
| Ffriddoedd site-Reichel | 10/01/2022 15:00 | 10/01/2022 09:30 | 10/01/2022 15:00 |  |  | 4466413 | 1324 | 0 | 0 | 0 | 0 | 0 |  | 0 |  |  | 0 |
| St Mary's | 12/01/2022 15:00 | 12/01/2022 09:00 | 12/01/2022 15:00 |  |  | 1928613 | 3531 | 0 | 0 | 0 | 0 | 0 |  | 0 |  |  | 1225.46 |
| Ffriddoedd site-Road | 12/01/2022 15:10 | 12/01/2022 09:00 | 12/01/2022 15:10 |  |  | 1815408 | 4217 | 0 | 0 | 0 | 0 | 0 |  | 0 |  |  | 966.21 |
| Ffriddoedd site-Brailsford | 12/01/2022 15:20 | 12/01/2022 09:00 | 12/01/2022 15:20 |  |  | 1372889 | 1576 | 0 | 0 | 0 | 0 | 0 |  | 0 |  |  | 391.82 |
| Ffriddoedd site-Reichel | 12/01/2022 15:30 | 12/01/2022 09:00 | 12/01/2022 15:30 |  |  | 2645855 | 52 | 0 | 0 | 0 | 0 | 0 |  | 0 |  |  | 0 |
| St Mary's | 13/01/2022 09:00 | 12/01/2022 15:00 | 13/01/2022 09:00 |  |  | 4287645 | 27319 | 0 | 0 | 138479.2 | 0 | 0 |  | 0 |  |  | 5090.08 |
| Ffriddoedd site-Road | 13/01/2022 09:10 | 12/01/2022 15:10 | 13/01/2022 09:10 |  |  | 3428734 | 76 | 0 | 0 | 0 | 0 | 0 |  | 0 |  |  | 0 |
| Ffriddoedd site-Brailsford | 13/01/2022 09:20 | 12/01/2022 15:20 | 13/01/2022 09:20 |  |  | 2794521 | 103 | 0 | 0 | 0 | 0 | 0 |  | 0 |  |  | 0 |
| Ffriddoedd site-Reichel | 13/01/2022 09:30 | 12/01/2022 15:30 | 13/01/2022 09:30 |  |  | 1165906 | 149 | 0 | 0 | 0 | 0 | 0 |  | 0 |  |  | 0 |
| St Mary's | 13/01/2022 15:00 | 13/01/2022 09:00 | 13/01/2022 15:00 | 0.1512 |  | 11805131 | 31922 | 0 | 0 | 44669.95 | 0 | 0 |  | 0 |  |  | 10803.87 |
| Ffriddoedd site-Road | 13/01/2022 15:10 | 13/01/2022 09:10 | 13/01/2022 15:10 | 0.1831 |  | 7985782 | 92 | 0 | 0 | 0 | 0 | 0 |  | 0 |  |  | 0 |
| Ffriddoedd site-Brailsford | 13/01/2022 15:20 | 13/01/2022 09:20 | 13/01/2022 15:20 | 0.2253 |  | 12274921 | 1398 | 0 | 0 | 0 | 0 | 0 |  | 0 |  |  | 0 |
| Ffriddoedd site-Reichel | 13/01/2022 15:30 | 13/01/2022 09:30 | 13/01/2022 15:30 | 0.1865 |  | 1317213 | 9296 | 0 | 0 | 0 | 0 | 0 |  | 0 |  |  | 2301.75 |
| St Mary's | 14/01/2022 08:00 | 13/01/2022 15:00 | 14/01/2022 08:00 | 0.1284 |  | 6368495 | 24508 | 0 | 0 | 144368.9 | 0 | 0 |  | 0 |  |  | 7159.7 |
| Ffriddoedd site-Road | 14/01/2022 08:10 | 13/01/2022 15:10 | 14/01/2022 08:10 | 0.0896 | 0.0859 | 5300010 | 135 | 0 | 0 | 0 | 0 | 0 |  | 0 |  |  | 0 |
| Ffriddoedd site-Brailsford | 14/01/2022 08:20 | 13/01/2022 15:20 | 14/01/2022 08:20 | 0.2465 |  | 4906030 | 80 | 0 | 0 | 0 | 0 | 0 |  | 0 |  |  | 0 |
| Ffriddoedd site-Reichel | 14/01/2022 08:30 | 13/01/2022 15:30 | 14/01/2022 08:30 | 0.1827 |  | 3696495 | 33422 | 0 | 0 | 0 | 0 | 0 |  | 0 |  |  | 8913.91 |
| St Mary's | 14/01/2022 14:00 | 14/01/2022 08:00 | 14/01/2022 14:00 | 0.1562 |  | 12963584 | 5603 | 0 | 0 | 210550.3 | 0 | 0 |  | 0 |  |  | 1094.1 |
| Ffriddoedd site-Road | 14/01/2022 14:10 | 14/01/2022 08:10 | 14/01/2022 14:10 | 0.2130 |  | 4858266 | 172 | 0 | 0 | 0 | 0 | 0 |  | 0 |  |  | 0 |
| Ffriddoedd site-Brailsford | 14/01/2022 14:20 | 14/01/2022 08:20 | 14/01/2022 14:20 | 0.1375 |  | 6443427 | 44 | 0 | 0 | 0 | 0 | 0 |  | 0 |  |  | 0 |
| Ffriddoedd site-Reichel | 14/01/2022 14:30 | 14/01/2022 08:30 | 14/01/2022 14:30 | 0.1075 |  | 8552185 | 7647 | 0 | 0 | 0 | 0 | 0 |  | 0 |  |  | 1832.13 |
| St Mary's | 17/01/2022 15:00 | 17/01/2022 09:00 | 17/01/2022 15:00 | 0.1011 |  | 1508669 | 961 | 0 | 0 | 29362.51 | 0 | 0 |  | 0 | 0 |  | 0 |
| Ffriddoedd site-Road | 17/01/2022 15:10 | 17/01/2022 09:10 | 17/01/2022 15:10 | 0.1871 |  | 3952425 | 1148 | 0 | 0 | 0 | 0 | 0 |  | 0 | 0 |  | 0 |
| Ffriddoedd site-Brailsford | 17/01/2022 15:20 | 17/01/2022 09:20 | 17/01/2022 15:20 | 0.0836 |  | 2784428 | 216553 | 0 | 0 | 0 | 0 | 0 |  | 4086.42 | 42859.23 |  | 35322.62 |
| Ffriddoedd site-Reichel | 17/01/2022 15:30 | 17/01/2022 09:30 | 17/01/2022 15:30 | 0.8449 |  | 2349666 | 40230 | 0 | 0 | 0 | 0 | 0 |  | 0 | 3347.3 |  | 6667.75 |
| Ffriddoedd site-Road | 18/01/2022 09:10 | 17/01/2022 15:30 | 18/01/2022 09:10 | 0.1480 |  | 877415 | 23312 | 0 | 0 | 0 | 0 | 0 |  | 0 | 0 |  | 3680.69 |
| Ffriddoedd site-Brailsford | 18/01/2022 09:20 | 17/01/2022 15:30 | 18/01/2022 09:20 | 0.1155 |  | 1767246 | 25416 | 0 | 0 | 0 | 0 | 0 |  | 1855.44 | 2214.01 |  | 6406.86 |
| Ffriddoedd site-Reichel | 18/01/2022 09:30 | 17/01/2022 15:30 | 18/01/2022 09:30 | 0.4203 |  | 939331 | 4391 | 0 | 0 | 0 | 0 | 0 |  | 0 | 0 |  | 0 |
| St Mary's | 18/01/2022 15:00 | 18/01/2022 09:00 | 18/01/2022 15:00 | 0.3197 |  | 3327221 | 1766 | 0 | 0 | 778.62 | 0 | 0 |  | 0 | 0 |  | 0 |
| Ffriddoedd site-Road | 18/01/2022 15:10 | 18/01/2022 09:10 | 18/01/2022 15:10 | 0.9396 |  | 2159366 | 2326 | 0 | 0 | 0 | 0 | 0 |  | 0 | 0 |  | 0 |
| Ffriddoedd site-Brailsford | 18/01/2022 15:20 | 18/01/2022 09:20 | 18/01/2022 15:20 | 0.0228 |  | 2522997 | 18402 | 0 | 0 | 0 | 0 | 0 |  | 506.3 | 0 |  | 629.35 |
| Ffriddoedd site-Reichel | 18/01/2022 15:30 | 18/01/2022 09:30 | 18/01/2022 15:30 | 0.2552 |  | 793901 | 7820 | 0 | 0 | 0 | 0 | 0 |  | 0 | 0 |  | 365.82 |
| St Mary's | 19/01/2022 09:00 | 18/01/2022 15:00 | 19/01/2022 09:00 | 0.0691 |  | 11000290 | 420 | 0 | 0 | 6536.6 | 0 | 0 |  | 0 | 0 |  | 0 |
| Ffriddoedd site-Road | 19/01/2022 09:10 | 18/01/2022 15:10 | 19/01/2022 09:10 | 0.2214 |  | 2003066 | 1255 | 0 | 0 | 0 | 0 | 0 |  | 0 | 0 |  | 0 |
| Ffriddoedd site-Brailsford | 19/01/2022 09:20 | 18/01/2022 15:20 | 19/01/2022 09:20 | 0.5692 |  | 2203875 | 9522 | 0 | 0 | 0 | 0 | 0 |  | 1011.66 | 0 |  | 0 |
| Ffriddoedd site-Reichel | 19/01/2022 09:30 | 18/01/2022 15:30 | 19/01/2022 09:30 | 0.0399 |  | 1406484 | 4759 | 0 | 0 | 0 | 0 | 0 |  | 0 | 0 |  | 902.39 |
| St Mary's | 19/01/2022 15:00 | 19/01/2022 09:00 | 19/01/2022 15:00 | 0.0568 |  | 11213059 | 250 | 0 | 0 | 0 | 0 | 0 |  | 0 | 0 |  | 0 |
| Ffriddoedd site-Road | 19/01/2022 15:00 | 19/01/2022 09:10 | 19/01/2022 15:00 | 0.4107 |  | 36237378 | 846 | 0 | 0 | 0 | 0 | 0 |  | 0 | 0 |  | 368.19 |
| Ffriddoedd site-Brailsford | 19/01/2022 15:00 | 19/01/2022 09:20 | 19/01/2022 15:00 | 0.5483 |  | 7716128 | 5907 | 0 | 0 | 0 | 0 | 0 |  | 251.37 | 10139.99 |  | 2278.97 |
| Ffriddoedd site-Reichel | 19/01/2022 15:00 | 19/01/2022 09:30 | 19/01/2022 15:00 | 0.4413 |  | 35227693 | 80484 | 0 | 0 | 0 | 0 | 0 |  | 0 | 123624.9 |  | 43525.55 |
| St Mary's | 21/01/2022 09:00 | 19/01/2022 15:00 | 21/01/2022 09:00 | 0.0020 |  | 8733551 | 714 | 0 | 0 | 0 | 0 | 0 |  | 0 | 0 |  | 115.37 |
| Ffriddoedd site-Road | 21/01/2022 09:10 | 19/01/2022 15:10 | 21/01/2022 09:10 | 0.1383 |  | 3230629 | 2409 | 0 | 0 | 0 | 0 | 0 |  | 0 | 0 |  | 559.4 |
| Ffriddoedd site-Brailsford | 21/01/2022 09:20 | 19/01/2022 15:20 | 21/01/2022 09:20 | 0.0370 |  | 7715944 | 3200 | 0 | 0 | 0 | 0 | 0 |  | 482.78 | 0 |  | 307.35 |
| Ffriddoedd site-Reichel | 21/01/2022 09:30 | 19/01/2022 15:30 | 21/01/2022 09:30 | 0.1452 |  | 11223680 | 1076 | 0 | 0 | 0 | 0 | 0 |  | 0 | 0 |  | 190.53 |
| St Mary's | 21/01/2022 15:00 | 21/01/2022 09:00 | 21/01/2022 15:00 | 0.7455 |  | 1084584 | 315 | 0 | 0 | 0 | 0 | 0 |  | 0 | 0 |  | 0 |
| Ffriddoedd site-Road | 21/01/2022 15:10 | 21/01/2022 09:10 | 21/01/2022 15:10 | 7.0475 |  | 649261 | 174 | 0 | 0 | 0 | 0 | 0 |  | 0 | 0 |  | 0 |
| Ffriddoedd site-Brailsford | 21/01/2022 15:20 | 21/01/2022 09:20 | 21/01/2022 15:20 | 0.1245 |  | 2057030 | 1528 | 0 | 0 | 0 | 0 | 0 |  | 0 | 0 |  | 0 |
| Ffriddoedd site-Reichel | 21/01/2022 15:30 | 21/01/2022 09:30 | 21/01/2022 15:30 | 1.5300 |  | 1930798 | 1281 | 0 | 0 | 0 | 0 | 0 |  | 0 | 0 |  | 0 |
| St Mary's | 24/01/2022 15:00 | 24/01/2022 09:00 | 24/01/2022 15:00 | 0.0955 |  | 3579619 | 252 | 0 | 0 | 630896.7 | 0 | 0 |  | 0 | 0 |  | 0 |
| Ffriddoedd site-Road | 24/01/2022 15:10 | 24/01/2022 09:10 | 24/01/2022 15:10 | 0.0248 |  | 4446462 | 2980 | 0 | 0 | 0 | 0 | 0 |  | 0 | 0 |  | 139.57 |
| Ffriddoedd site-Brailsford | 24/01/2022 15:20 | 24/01/2022 09:20 | 24/01/2022 15:20 | 0.0369 |  | 1440062 | 715 | 0 | 0 | 0 | 0 | 0 |  | 0 | 0 |  | 42.73 |
| Ffriddoedd site-Reichel | 24/01/2022 15:30 | 24/01/2022 09:30 | 24/01/2022 15:30 | 0.2977 |  | 1821392 | 4564 | 0 | 0 | 0 | 0 | 0 |  | 0 | 0 |  | 201.99 |
| St Mary's | 26/01/2022 15:00 | 26/01/2022 09:00 | 26/01/2022 15:00 | 0.1106 |  | 8099689 | 750 | 0 | 0 | 0 | 0 | 0 |  | 0 | 0 |  | 191.35 |
| Ffriddoedd site-Road | 26/01/2022 15:10 | 26/01/2022 09:00 | 26/01/2022 15:10 |  |  |  |  |  |  |  |  |  |  |  |  |  |  |
| Ffriddoedd site-Brailsford | 26/01/2022 15:20 | 26/01/2022 09:00 | 26/01/2022 15:20 | 0.0250 |  | 27943324 | 399 | 0 | 0 | 0 | 0 | 0 |  | 0 | 0 |  | 61.91 |
| Ffriddoedd site-Reichel | 26/01/2022 15:30 | 26/01/2022 09:00 | 26/01/2022 15:30 | 0.2124 |  | 48417529 | 354 | 0 | 0 | 0 | 0 | 0 |  | 0 | 0 |  | 96.02 |

**References**

1. Centers for Disease Control and Prevention. 2020. Real-time RT-PCR Primers and Probes for COVID-19. Centers for Disease Control and Prevention. https://www.cdc.gov/coronavirus/2019-ncov/lab/rt-pcr-panel-primer-probes.html. Retrieved 28 January 2022.

2. Gendron L, Verreault D, Veillette M, Moineau S, Duchaine C. 2010. Evaluation of filters for the sampling and quantification of RNA phage aerosols. Aerosol Science and Technology 44:893–901.

3. Shu B, Kirby MK, Davis WG, Warnes C, Liddell J, Liu J, Wu KH, Hassell N, Benitez AJ, Wilson MM, Keller MW, Rambo-Martin BL, Camara Y, Winter J, Kondor RJ, Zhou B, Spies S, Rose LE, Winchell JM, Limbago BM, Wentworth DE, Barnes JR. 2021. Multiplex Real-Time Reverse Transcription PCR for Influenza A Virus, Influenza B Virus, and Severe Acute Respiratory Syndrome Coronavirus 2. Emerg Infect Dis 27:1821.

4. Poelman R, Schölvinck EH, Borger R, Niesters HGM, Van Leer-Buter C. 2015. The emergence of enterovirus D68 in a Dutch University Medical Center and the necessity for routinely screening for respiratory viruses. Journal of Clinical Virology 62:1.

5. Stachler E, Kelty C, Sivaganesan M, Li X, Bibby K, Shanks OC. 2017. Quantitative crAssphage PCR assays for human fecal pollution measurement. Environ Sci Technol 51:9146–9154.
